# Orthogonal Enzyme-Driven Timers for DNA Strand Displacement Reactions

**DOI:** 10.1021/jacs.2c06599

**Published:** 2022-10-18

**Authors:** Juliette Bucci, Patrick Irmisch, Erica Del Grosso, Ralf Seidel, Francesco Ricci

**Affiliations:** † Chemistry Department, University of Rome, Tor Vergata, Via della Ricerca Scientifica, 00133 Rome, Italy; ‡ Molecular Biophysics Group, Peter Debye Institute for Soft Matter Physics, Universität Leipzig, 04103 Leipzig, Germany

## Abstract

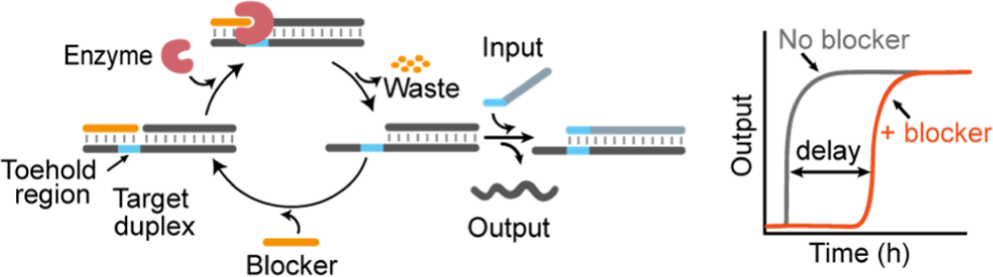

Here, we demonstrate a strategy to rationally program a delayed onset of toehold-mediated DNA strand displacement reactions (SDRs). The approach is based on blocker strands that efficiently inhibit the strand displacement by binding to the toehold domain of the target DNA. Specific enzymatic degradation of the blocker strand subsequently enables SDR. The kinetics of the blocker enzymatic degradation thus controls the time at which the SDR starts. By varying the concentration of the blocker strand and the concentration of the enzyme, we show that we can finely tune and modulate the delayed onset of SDR. Additionally, we show that the strategy is versatile and can be orthogonally controlled by different enzymes each specifically targeting a different blocker strand. We designed and established three different delayed SDRs using RNase H and two DNA repair enzymes (formamidopyrimidine DNA glycosylase and uracil-DNA glycosylase) and corresponding blockers. The achieved temporal delay can be programed with high flexibility without undesired leak and can be conveniently predicted using kinetic modeling. Finally, we show three possible applications of the delayed SDRs to temporally control the ligand release from a DNA nanodevice, the inhibition of a target protein by a DNA aptamer, and the output signal generated by a DNA logic circuit.

## Introduction

Predictable base-pairing, low-cost production, and chemical versatility make synthetic DNA an optimal material to build nanoscale objects and devices that can find applications in fields like sensing, drug delivery, and therapeutics.^1−4^ The majority of these systems rely on simple programmable reactions between synthetic DNA strands that allow us to create well-defined static and dynamic two-dimensional or three-dimensional structures.^5−7^ Several strategies have been proposed to date to make such DNA-based systems responsive to different molecular and environmental inputs that include proteins, small molecules, pH, and temperature.^8−12^ Spatial reconfigurations and precise input-induced conformational changes have also been demonstrated to control the functionality of the DNA-based systems.^13−17^ Beyond this, significant efforts have been devoted to establish reaction networks that can carry out signal processing and dynamic signal generation in analogy to biological systems.^18−22^ Living systems and cellular pathways are not only precisely controlled by environmental and molecular cues but are also temporally programed using elaborate positive and negative feedback mechanisms that typically operate through out-of-equilibrium, dissipative, or delayed reactions.^23−26^ Inspired by this, several DNA-based reaction systems have been reported to date whose dynamics can be controlled in a programmable way, for example, by altering the energy landscapes of the process,^27−30^ by implementing negative/positive feedback loops,^31,32^ and by employing dissipative reaction steps.^33−38^ This allowed us to set up synthetic reaction networks with a complex time-dependent behavior that in parallel can modulate the functionality and/or assembly of DNA downstream systems.^39−46^

When controlling the time dependence of reaction systems, an important function are tunable delays of individual reactions, for example, when establishing feedback loops. Some attempts have been made to obtain the DNA-based reactions with tunable delays. Examples include the design of DNA-based circuits that sequentially release different DNA sequences from sequestered complexes to achieve a sustained release of a DNA strand with a tunable delay.^47,48^ Self-amplification systems have also been exploited to introduce delays in DNA-based reactions, though leak reactions limit the temporal modulation in this case.^49^ Finally, ATP-dependent enzymatic reaction networks were also programed to temporally control the assembly of DNA-based polymers and structures.^33,50^

**Figure 1:**
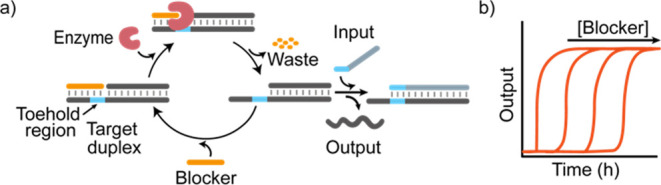
(a) Scheme of the enzyme-driven delay for SDRs. A blocker strand (orange) bound to the toehold region (light blue) of the DNA target strand initially inhibits input binding and displacement of the output strand. Specific degradation of the blocker by an enzyme during the course of the reaction frees the toehold and allows SDR after a delay. (b) The delay time at which the reaction starts can be tuned by modulating the kinetics of blocker degradation using different concentrations of enzyme or blocker.

Though the above examples clearly demonstrate the utility of temporal delays within DNA-based reaction systems, a tight and independent orchestration of a series of reaction events within reaction networks would require simpler and more robust molecular timers that could operate in parallel. Here, we tackle this problem and report an enzyme-based strategy to temporally control DNA-based reactions. Core of our approach is the control of the onset of toehold-mediated (or toehold-exchange) DNA strand displacement reactions (SDRs). This type of reaction, in which an input strand displaces an output strand that is hybridized to a complementary target strand, is widely employed in the field of DNA nanotechnology and has been used to precisely control the assembly/disassembly of DNA structures and the operation of DNA-based devices.^14,16,51−56^ SDR is promoted by the binding of the input strand to a single-stranded toehold domain on the target duplex.^54^ In a typical SDR, rapid output displacement starts directly after input addition. To establish a tunable delay, we propose here the use of *blocker* strands that hybridize to the toehold region of the target duplex and can be enzymatically degraded over time. Thus, while initially input strand binding and strand displacement is inhibited due to the presence of the blocker, it is subsequently enabled after a delay that is determined by the kinetics of the blocker degradation reaction (Figure 1). The delayed onset of the strand displacement can be easily tuned over a large time period by varying the concentrations of the blocker strand or the enzyme. Moreover, we identify different enzyme-blocker couples to design fully orthogonal systems in which multiple SDRs can be delayed in parallel.

## Results and Discussion

**Figure 2:**
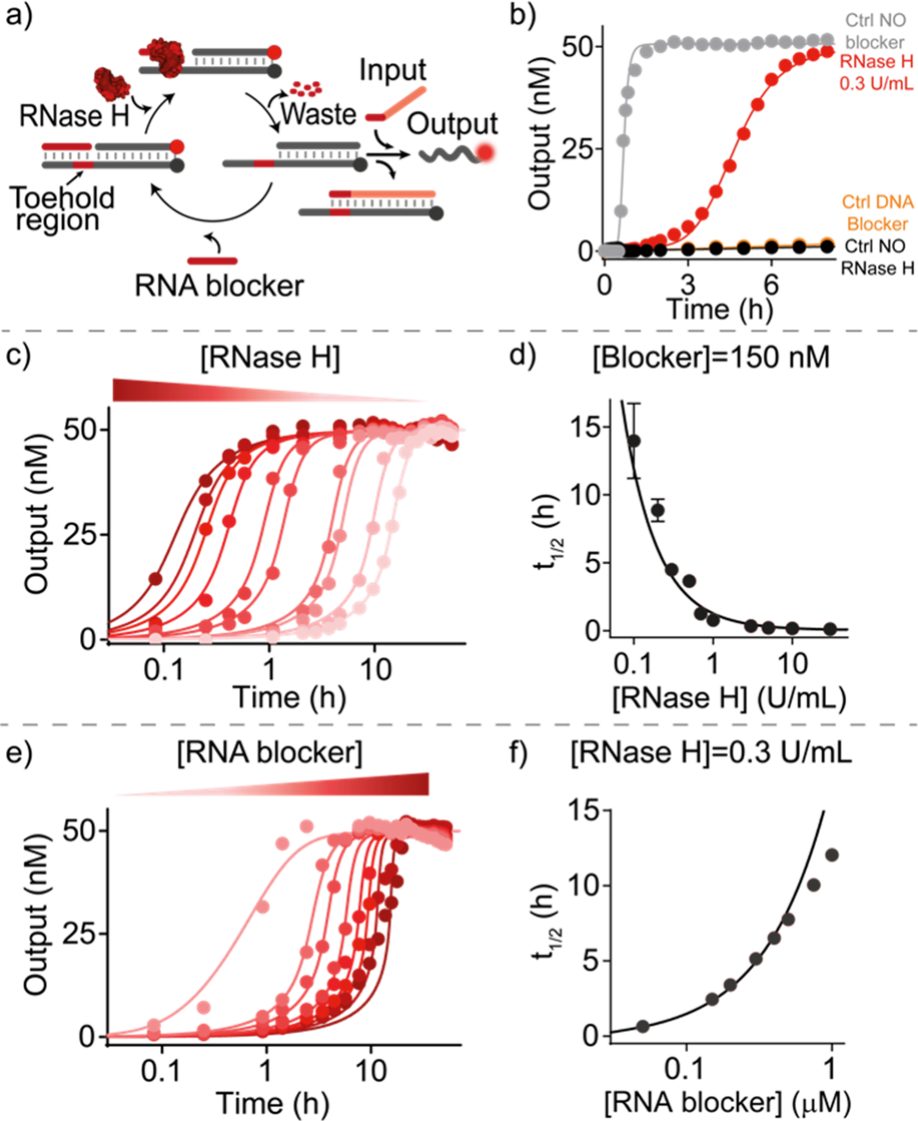
Delayed SDR using an RNA-blocker and RNase H. (a) Scheme of the reaction. RNA-blocked toeholds can be liberated by enzymatic blocker degradation and re-blocked. Only after blocker consumption, SDR is activated. (b) Time-course experiments of delayed SDR (red trace) and control experiments (without a blocker strand, gray trace; without RNase H, black trace; with a DNA blocker, orange trace). (c) Time-course experiments of SDR in the presence of the RNA blocker (150 nM) after the addition of different concentrations of RNase H (from 30 to 0.1 U/mL). (d) Reaction half-life as a function of the RNase H concentration. (e) Time-course experiments of SDR in the presence of RNase H (0.3 U/mL) and varying concentrations of the RNA blocker (from 0.05 to 1 μM). (f) Reaction half-life as a function of the RNA-blocker concentration. In (c–f), experimental values (dots) are shown together with fits (c,e) and prediction (d,f) from a parameterized kinetic model (solid lines) described in section 2b of Supporting Information. Shown experiments were performed in Tris HCl 20 mM, MgCl_2_ 10 mM, ethylenediaminetetraacetic acid (EDTA) 1 mM, pH 8.0 at *T* = 30 °C, using 50 nM target duplex and 50 nM input strand. In (b), the RNA (or DNA) blocker strand concentration was 150 nM.

As a first attempt to establish a tunable delay for DNA SDR based on enzymatic degradation of a toehold blocker, we employed an RNA strand as the blocker and the endoribonuclease RNase H as the corresponding blocker-degrading enzyme. RNase H is active only within DNA/RNA heteroduplexes; that is, blocker strands are specifically degraded when bound to the toehold.^57^ The DNA target duplex displays a 20-bp double-stranded region and a single-stranded overhang of 20 nucleotides that serves as the binding domain for the RNA-blocker strand and as the toehold domain (only the first 5 nucleotides) for the input strand (Figure 2a). The target duplex is labeled with a fluorophore/quencher pair so that the strand displacement can be easily monitored in real time by the increasing fluorescence signal from the displaced output (Figure 2a). The reactions were carried out at a fixed concentration of target duplex (50 nM) and input (50 nM). In the absence of the RNA-blocker strand, the displacement reaction proceeds rapidly upon the addition of the input strand with a reaction half-life (*t*_1/2_) (i.e., time required to achieve 50% strand displacement) of 3.0 ± 0.7 min (Figure 2b, gray trace). In contrast, in the presence of the blocker, we observe a complete inhibition of the SDR that, even after 18 h, does not show any significant leakage (Figures 2b and S1b, black trace). This demonstrates that the blocker efficiently prevents the input from binding and inducing SDR. When adding RNase H to the reaction mixture, the blocking is relieved after a certain delay (Figure 2b, red trace). As a further control experiment, we have employed a DNA blocker that, even in the presence of RNase H, does not allow the SD reaction to occur (Figure 2b, orange trace). The effective delay of SDR achieved with RNase H and the RNA-blocker strand would depend on the blocker degradation rate. For example, delay modulation could be achieved at a fixed concentration of the RNA-blocker and varying the RNase H concentration. To test this idea, we carried out displacement reactions using a fixed concentration of the target duplex, input strand, and RNA-blocker and varying concentrations of RNase H (Figure 2c). In agreement with our expectation, the delay increases strongly upon decreasing enzyme concentration: observed *t*_1/2_ values vary from 0.11 ± 0.01 to 14 ± 2 h when changing the RNase H concentration from 30 to 0.1 U/mL (Figure 2d). In a similar way, the delay of the reaction can also be efficiently modulated by varying the concentration of the RNA-blocker at a fixed concentration of enzyme. By doing so, we were able to modulate *t*_1/2_ values from 0.64 ± 0.02 to 12.0 ± 0.2 h by varying the concentration of the blocker from 0.05 to 1.0 μM, respectively (Figure 2e,f). The delayed SDR was also confirmed by native polyacrylamide gel electrophoresis (PAGE) experiments and fluorescence emission spectra (Supporting Information, Figures S2 and S3).

To better understand the accomplished delayed strand displacement, we modeled the observed kinetics using a minimalistic reaction pathway. Essentially, blocked toeholds can be liberated by enzymatic blocker degradation and either re-blocked as long as blocker strands are not consumed or used for downstream irreversible SDRs by the input strand (Figures S4 and S5, see Supporting Information Section 2a,b for more details on the model). Modeling the experimental data provides a very good agreement with our kinetic model over the entire range of the blocker and enzyme concentrations tested (Figure 2c–f, solid lines). This supports our mechanistic understanding of the accomplished delays and demonstrates that the undesired leak reaction pathways play a minor role in the observed kinetics. Furthermore, it provides the essential rate constants of the reaction sub-steps (see Supporting Information, Section 2d).

**Figure 3:**
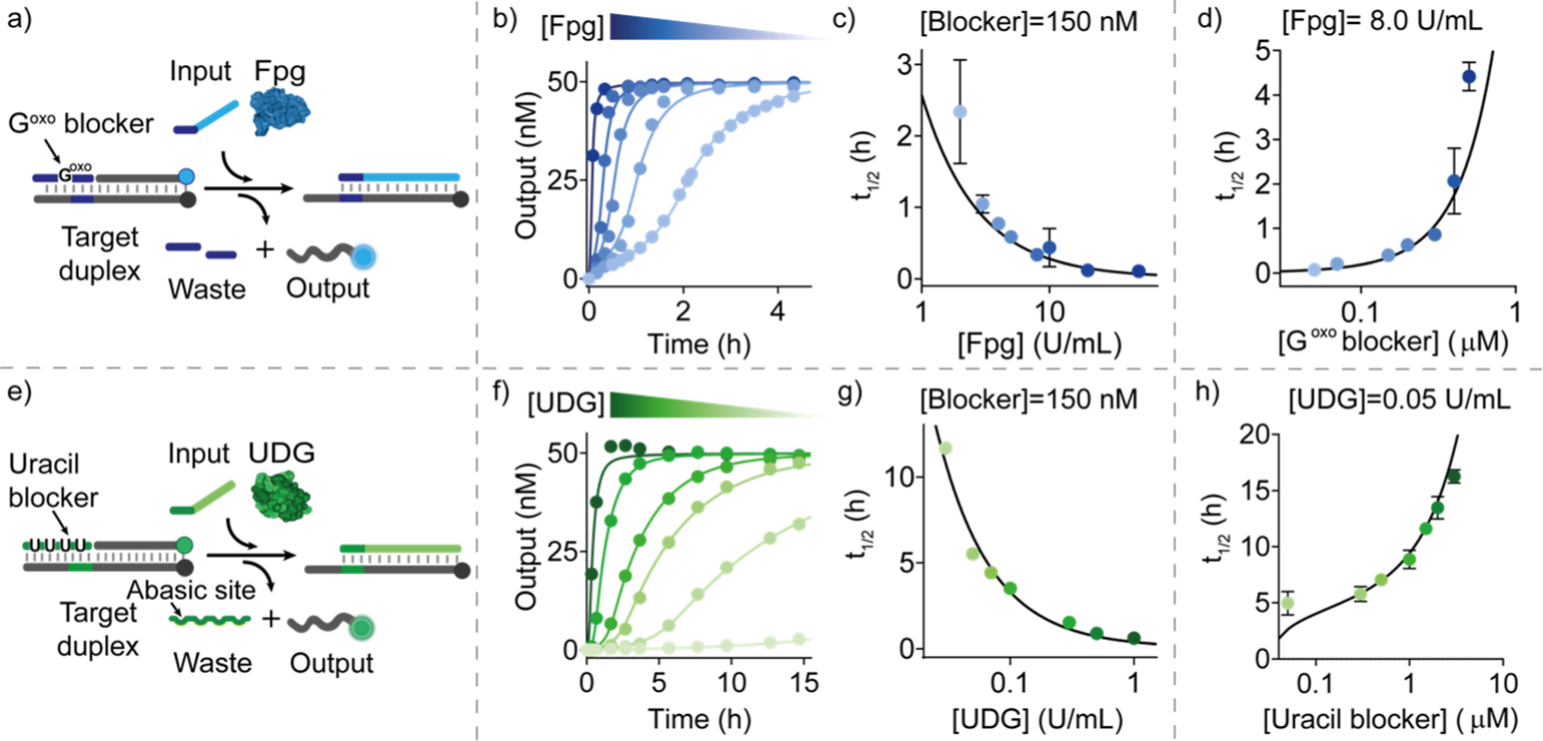
Delayed SDR using Fpg and UDG enzymes. (a) Scheme showing temporal control of SDR using the enzyme Fpg and a blocker strand containing a modified G^oxo^ base in its center. (b) Time-course experiments in the presence of the G^oxo^-blocker (150 nM) after the addition of different concentrations of Fpg. (c) Half-life values vs Fpg concentrations at a fixed concentration of the G^oxo^-blocker strand (150 nM). (d) Half-life values vs G^oxo^-blocker concentrations at a fixed concentration of Fpg (8.0 U/mL). (e) Scheme showing temporal control of SDR using the enzyme UDG and a blocker strand containing uracil bases. (f) Time-course experiments in the presence of the uracil-blocker (150 nM) after the addition of different concentrations of UDG. (g) Half-life values vs UDG concentrations at a fixed concentration of the uracil-blocker strand (150 nM). (h) Half-life values vs uracil blocker concentrations at a fixed concentration of UDG (0.05 U/mL). In (b–d) and (f–h), experimental values (dots) are shown together with fits (b,f) and prediction (c,d,g,h) from a parameterized kinetic model (solid lines) described in section 2e,f of Supporting Information. Experiments were performed in Tris HCl 20 mM, MgCl_2_ 10 mM, EDTA 1 mM, pH 8.0 at *T* = 30 °C. [Target duplex] = 50 nM, [input strand] = 50 nM.

To allow a programmable orchestration of reaction events, we next set out to develop alternative systems that allow for tunable delays using different enzymes and toehold blockers. In one system, we employed for blocker degradation the DNA repair enzyme formamidopyrimidine DNA glycosylase (Fpg). This enzyme cleaves DNA strands containing the modified base 8-oxo-7,8 dihydroguanine (G^oxo^) creating a 1 nt DNA gap within the phosphate backbone.^58^ As the blocker strand, we thus designed a 16 nt DNA strand containing a single G^oxo^ in its center. Upon enzymatic cleavage, two short fragments (8 and 7 nucleotides) are obtained that would spontaneously dissociate from the target duplex and either enable re-blocking by a new blocker or the initiation of SDR by the input strand (Figure 3a). As for the previous system, first we demonstrate that the G^oxo^-blocker strand is able to completely inhibit SDR (Figure S6b, black trace). Only after the addition of Fpg, SDR is unlocked due to the degradation of the G^oxo^-blocker strand that liberates the toehold domain (Figure S6b, light blue trace). Also in this case, a control experiment using a DNA blocker strand (without modified bases) demonstrates the specificity of the blocker degradation reaction (Figure S6b, orange trace). Native PAGE electrophoresis experiments (Figure S7) and emission spectra (Figure S8) support the above results.

Also with this system, we were able to obtain delayed sigmoidal curves in the presence of the blocker strand and Fpg (Figure 3b) similar to the ones previously obtained with the RNA/RNase H system. To tune the delay of reactions, we thus carried out SDRs at different concentrations of the G^oxo^-blocker and Fpg enzyme. For example, when fixing the concentration of the blocker strand to 150 nM, *t*_1/2_ increases from 0.11 ± 0.02 to 2.3 ± 0.7 h by decreasing the Fpg concentration from 50.0 to 2.0 U/mL, respectively (Figures 3b,c and S6c). Similarly, when increasing the concentration of the blocker strand from 0.05 to 0.50 μM at a fixed concentration of Fpg (8 U/mL), *t*_1/2_ increases from 0.070 ± 0.005 h to 4.4 ± 0.3 h, respectively (Figures 3d and S6d). Using a slightly adapted reaction scheme (see Supporting Information, Section 2e), we are also in this case able to accurately describe the obtained reaction kinetics for the different conditions (Figure 3b–d, solid lines) and to derive the essential rate constants of the reaction sub-steps (see Supporting Information, Section 2e).

As a third possibility to set up tunable delays, we employed uracil-DNA glycosylase (UDG), a base-excision repair enzyme that hydrolyzes deoxyuridine mutations from ssDNA or dsDNA strands, leading to the formation of abasic sites.^59^ In this case, we used a DNA blocker strand containing 4 deoxyuridine mutations (uracil-blocker, green, Figure 3e). Upon formation of apurinic sites in the blocker strand by UDG, the blocker-target duplex becomes destabilized, enabling the spontaneous dissociation of the blocker strand and the activation of the downstream SDR. Also with this system, we first performed control experiments to demonstrate that in the absence of UDG, the uracil-blocker strand is able to inhibit SDR (Figure S9b, black trace). The reaction is unlocked, with a certain delay, only after the addition of UDG (Figure S9b, light green trace). Native PAGE electrophoresis experiments (Figure S10) and emission spectra (Figure S11) support the above results.

Also with this other system, we are able to precisely control the observed delays with *t*_1/2_ increasing from 0.61 ± 0.01 to 11.7 ± 0.2 h when decreasing the concentration of UDG from 1.0 to 0.03 U/mL while using a fixed concentration of the uracil-blocker (Figures 3f,g and S9c). Similarly, by increasing the concentration of the uracil-blocker from 0.05 to 3.0 μM (at a fixed concentration of UDG), we can modulate *t*_1/2_ from 5.0 ± 1.0 to 16.2 ± 0.6 h, respectively (Figures 3h and S9d). Using a slightly adapted reaction scheme (see Supporting Information, Section 2f), we are also in this case able to accurately describe the obtained reaction kinetics for the different conditions (Figure 3f–h, solid lines) and also to derive the essential rate constants of the reaction sub-steps (see Supporting Information, Section 2f).

**Figure 4:**
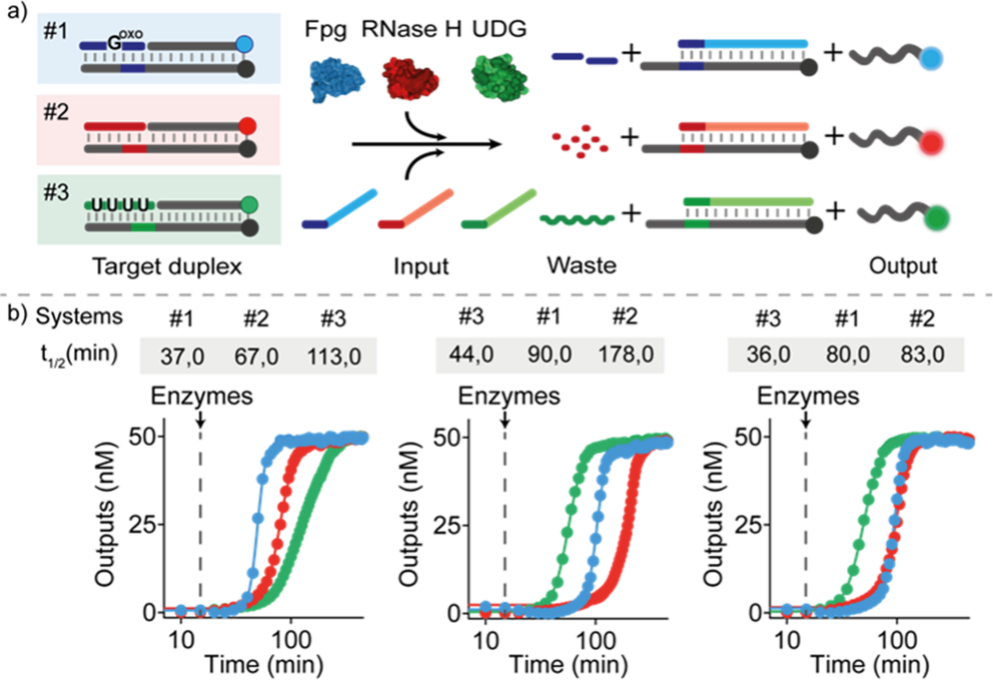
Orthogonal temporal control of SDRs. (a) Scheme showing the three orthogonal systems each controlled by a different enzyme/blocker couple and each labeled with a different fluorophore/quencher pair for orthogonal temporal control in the same solution. (b) Three examples of time-course experiments performed in one solution. Varying the enzyme concentrations shifts the order of activation of the three systems. Experiments were performed in Tris HCl 20 mM, MgCl_2_ 10 mM, EDTA 1 mM, pH 8 at *T* = 30 °C. [Target duplex] = 50 nM, [RNA-blocker] = 150 nM, [uracil-blocker] = 150 nM, [G^oxo^-blocker] = 250 nM, [input strand] = 50 nM (for each system). The following concentrations of enzymes were employed in the time-course experiments shown in panel b: left: Fpg 10.0 U/mL, RNase H 0.7 U/mL, UDG 0.05 U/mL; middle: Fpg 8.0 U/mL, RNase H 0.3 U/mL, UDG 0.3 U/mL; right: Fpg 8.0 U/mL, RNase H 0.5 U/mL, UDG 0.3 U/mL.

By combining the high programmability and selectivity of nucleic acid interaction with the specificity of enzymes, our approach allows us to temporally control multiple SDRs in the same solution. To demonstrate this, we performed time-course fluorescent experiments using the above three established enzyme systems in a one-pot reaction employing different combinations of blocker-enzyme concentrations for each system (Figure 4a). This allowed us to modulate the order of the onsets of the different displacement reactions in a programmable fashion. Of note, each system employs a different target duplex (each labeled with a specific fluorophore/quencher pair), a specific input strand, and a unique blocker-enzyme couple (Figure S12). As an example, we report three different experiments in which the three systems become independently activated at different times (Figure 4b). Further combinations were established by varying the concentration of either the blocker strand or enzyme of each system (Figure S13).

**Figure 5:**
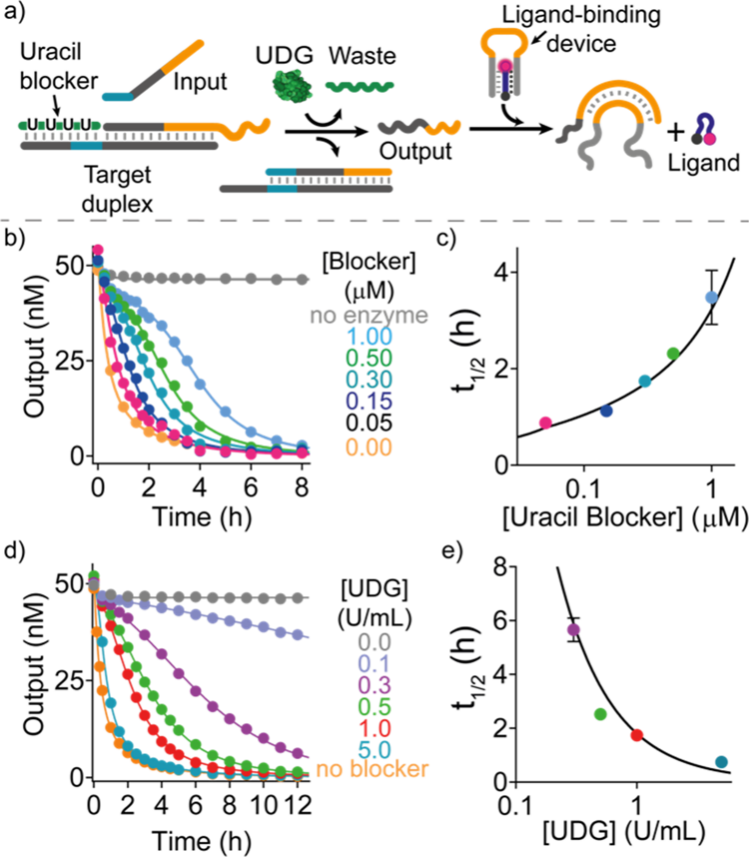
Temporally controlled ligand release from a DNA device. (a) Scheme of coupling delayed strand displacement to a DNA-based device that binds a 9 nt ligand DNA sequence by forming a triplex structure. The output strand of the delayed SDR binds the loop domain of the triplex structure and induces the ligand release. Tunable delays of SDR and thus of the downstream ligand release can be obtained in the presence of the blocker strand and its degrading enzyme UDG. (b) Time-course experiments using different concentrations of the uracil-blocker at a fixed concentration of UDG (1.0 U/mL). (c) Half-life values of ligand release obtained at different uracil-blocker concentrations. (d) Time-course experiments using different concentrations of UDG at a fixed concentration of the uracil-blocker (150 nM). (e) Half-life values of ligand release obtained at different UDG concentrations. In (b–e), experimental values (dots) are shown together with fits (b,d) and prediction (c,e) from a parameterized kinetic model (solid lines) described in Section 2g of Supporting Information. Experiments were performed in Tris HCl 10 mM, MgCl_2_ 3 mM, pH 6 at *T* = 25 °C. [Target duplex] = 50 nM, [uracil-blocker] = 150 nM, [ligand] = 50 nM, [input strand] = 50 nM, [ligand-binding device] = 50 nM.

We next tested whether our strategy can be readily applied to obtain tunable delays of downstream systems such as a DNA-based nanodevice. We first employed a DNA-based ligand-binding device^8,11,60^ that recognizes a specific 9 nt DNA sequence (ligand) through Watson–Crick and Hoogsteen interactions forming a stem-loop triplex structure. The binding of a DNA strand to the loop domain of this triplex structure causes a conformational change that induces the opening of the complex leading to the release of the DNA ligand from the device (Figure 5a). The activity of this DNA-based nanodevice is monitored by labeling the ligand strand with a fluorophore/quencher pair at the two ends. Upon ligand release, the distance between the two dyes decreases, leading to an observable decrease of the fluorescent signal. To achieve a tunable delay of ligand release, we designed a SDR in which the output strand is complementary to the loop domain of the device and can trigger the downstream ligand release from the nanodevice. The onset time of SDR can be programed using a uracil-blocker and UDG enzyme. We performed time-course fluorescent experiments in a solution containing an equimolar concentration (50 nM) of the ligand/device complex and target duplex and followed ligand release upon the addition of the input strand (50 nM). In the absence of the blocker strand and enzyme, as expected, we observe a rapid ligand release (signal decrease) with a *t*_1/2_ of 0.60 ± 0.01 h (Figure 5b, orange curve). When the same experiment is carried out in the presence of the blocker strand (150 nM), no ligand release is observed even after 8 h (Figure 5b, gray curve).

In the presence of the blocker and enzyme, a delayed ligand release is observed that can be tuned with by the reactant concentrations. At a fixed concentration of UDG, *t*_1/2_ values of the ligand release increase from 0.91 ± 0.06 to 3.90 ± 0.60 h when increasing the concentration of the uracil-blocker from 0.05 to 1.0 μM (Figure 5b,c). Similarly, upon decreasing the concentration of UDG from 5 to 0.1 U/mL at a fixed concentration of the uracil-blocker, the *t*_1/2_ values increase from 0.74 ± 0.01 to 5.6 ± 0.4 h, respectively (Figure 5d,e). To gain a deeper understanding of the observed kinetics, we modeled them by employing an extended reaction scheme for the UDG system by an additional ligand release step, used also to obtain the essential rate constants of the reaction sub-steps (see Supporting Information, Section 2g). Again, the model reproduces the experimental data with high accuracy (solid lines Figure 5b–e). Particularly, it describes the sigmoidal appearance of the curves indicating the successful establishment of the delay.

**Figure 6:**
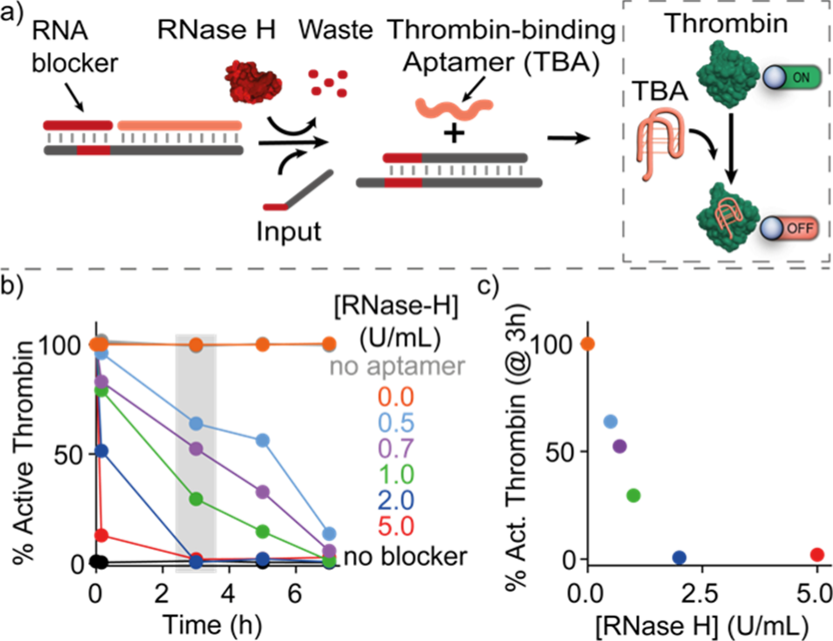
Temporal control of thrombin activity. (a) Scheme showing temporal control of thrombin activity regulated by an upstream delayed SDR mediated by RNase H. The output strand of this reaction is the G-quadruplex TBA that inhibits thrombin activity (dashed box). (b) Percentage of active thrombin VS time (from the addition of RNase H in solution) for different concentrations of RNase H. (c) Percentage of active thrombin after 3 h vs RNase H concentration. Experiments were performed in Tris HCl 20 mM, MgCl_2_ 10 mM, EDTA 1 mM, 150 mM NaCl, pH 8.0 at *T* = 25 °C. [Target duplex] = 50 nM, [RNA-blocker] = 150 nM, [thrombin] = 0.5 nM, [fibrinogen] = 1 mg/mL.

As an additional application, we explored whether the delayed strand displacement can be used to control and regulate the activity of a protein over time. We employed thrombin, a key enzyme involved in the blood coagulation cascade. The proteolytic cleavage activity of thrombin and the resulting transformation of soluble fibrinogen into insoluble fibrin can be specifically inhibited by a thrombin-binding aptamer (TBA), a single-stranded G-quadruplex-forming 15-mer DNA, that binds the thrombin fibrinogen-interacting site.^61^ To achieve temporal control over the activity of thrombin, we designed a RNase H-delayed SDR that releases as the output strand the TBA such that it would inhibit thrombin upon its release (Figure 6a). To follow the activity of thrombin in real time, we performed a light scattering assay in which the thrombin proteolytic conversion of fibrinogen to fibrin can be followed by the increase in light scattering. Only when adding the DNA input strand, which releases the aptamer by strand displacement, thrombin becomes successfully inhibited. This process is extremely rapid and, as a consequence, we observe complete inhibition of the thrombin activity within 10 min (Figure 6b, black curve). Conversely, when adding the RNA blocker (150 nM) to this reaction, no aptamer release is induced and no thrombin inhibition is obtained even after 7 h (Figure 6b, orange curve). By supplementing the reaction with different concentrations of RNase H, we can temporally control the thrombin activity. For example, by doing this, we show that the % of active thrombin after 3 h from the start of the reaction varies from 64.0 to 2.0% by varying the concentration of RNase H from 0.5 to 5.0 U/mL (Figure 6c).

**Figure 7:**
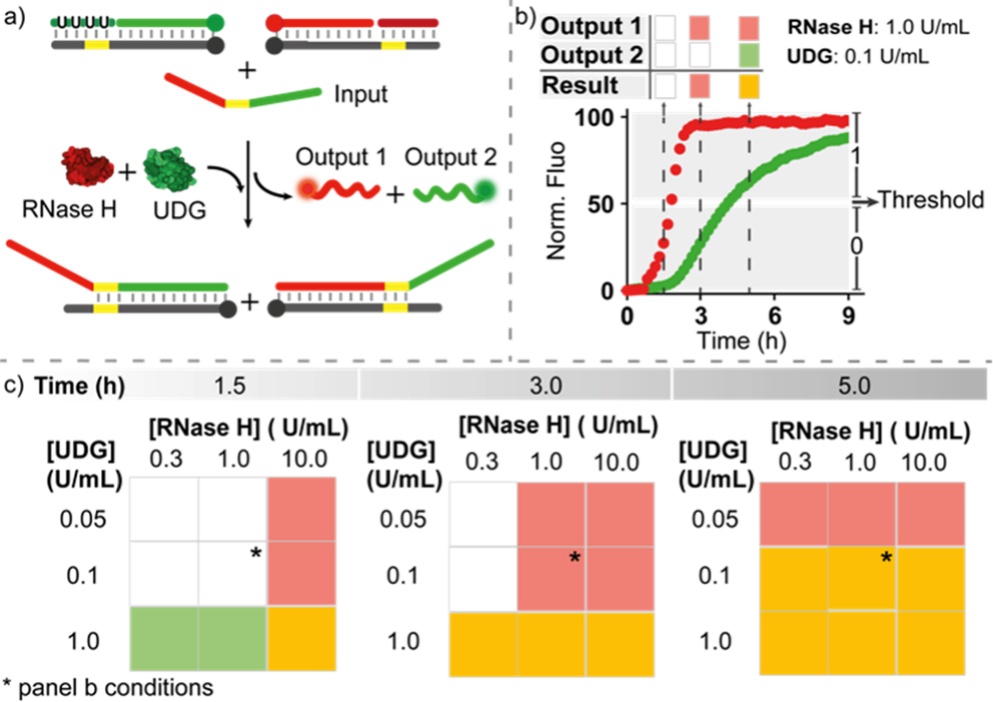
Enzyme-mediated temporal control of a DNA logic circuit. (a) Scheme of the logic circuit. (b) Example of a time-course experiment at the indicated concentrations of RNase H and UDG. (c) Two-dimensional decision matrix obtained at three representative times using different concentrations of RNase H and UDG. Experiments were performed in Tris HCl 20 mM, MgCl_2_ 10 mM, EDTA 1 mM, pH 8 at T = 30 °C. [Target duplexes] = 50 nM, [RNA-blocker] = 150 nM, [uracil-blocker] = 150 nM and at the indicated concentrations of RNase H and UDG.

As a third and final application, we demonstrate a DNA logic circuit, in which the delay of two different output strands, after addition of a single input strand, can be controlled independently using different blocker-degradation conditions (Figure 7a). To this end, we rationally designed two target duplexes that share the same toehold domain (yellow domains, Figure 7a) but have two different duplex sequences labeled with two orthogonal fluorophores. Further, we designed an input strand that contains a toehold-binding domain in the middle and is flanked by two invading domains (each specific for a different target duplex) (see Figure 7a). The toehold of each target duplex can be blocked by a different blocker strand (here, we employed an RNA-blocker and a uracil-blocker strand), such that the delay of strand displacement for the two systems can be controlled independently by two different enzymes (RNase H and UDG). Notably, the two output sequences can be freely chosen, such that they can interact with different downstream reaction pathways. To demonstrate that such logic circuit is behaving as desired, we performed time-course experiments using a fixed concentration of the input strand (100 nM) and of the two target duplexes (150 nM) and varying the concentration of the two enzymes (RNase H: 10.0; 1.0; 0.3 U/mL and UDG: 1.0; 0.1; 0.05 U/mL) (Figure 7b shows an example). We have set a threshold value at a signal corresponding to 50% of the maximum signal and provided the final result at three representative times (1.5, 3, and 5 h) (Figure 7c). The resulting 2-dimensional decision matrix allows us to evaluate at which point in time the particular reaction pathway is unlocked. As can be seen, our approach allows us to finely modulate the temporal onset of the two output signals in our logic circuit over multiple hours.

## Conclusions

Here, we developed a simple and robust strategy to establish molecular “timers” to determine the onset of toehold-mediated DNA SDRs. The essential components of our approach are blocker strands that protect the toehold domains. The toeholds become accessible only after a delay during which the blocker strands are enzymatically degraded such that programmable delays for strand displacement are obtained. Using three different enzymes (RNase H, Fpg, and UDG) acting on target-specific blocker strands, we established three different systems that can be operated in parallel in a fully orthogonal manner, that is, with an independent temporal control. Importantly, the output generation of our molecular “timers” can be conveniently coupled to different types of downstream reactions. More specifically, we demonstrated here three possible applications: (1) temporal control of ligand release from a DNA nanodevice, (2) protein inhibition by a DNA aptamer at programmable prespecified times, and (3) a DNA logic circuit in which temporal control of two independent outputs from a single input can be achieved. Of note, the delayed SDRs can be conveniently described by simple kinetic modeling that also allows us to predict the delay from the experimental conditions employed (concentration of the enzyme and blocker strand, temperature, etc.).

Regulating the timing of molecular events in DNA-based circuits is an important topic since it provides a basis for complex cascaded reaction networks that are capable of signal generation and processing. So far, the establishment of reaction networks concentrated predominantly on establishing the circuits based on Boolean logic as well as dynamic oscillators or dissipative transient responses.^36,44,62,63^ A significant fraction of these systems relies furthermore on amplification steps that are inherently leaky such that their applicability over a range of different time scales remains rather limited. Establishment of precise and versatile timers has therefore remained a challenge. Very recently, an elegant strategy was developed to establish a programed sequential output generation in cascaded SDRs.^48^ The usage of multiple sequential and clocked steps allowed a rather precise time shift of the output signal being close to a sigmoidal shape. Through avoiding signal amplification, the employed reaction scheme became quite complex and the output concentration decreased with each additional step.

In contrast, our approach employs only a simple blocker degradation reaction as an additional step in the conventional strand displacement process. Employing multi-turnovers of blocker strand degradation before the actual strand displacement allows us to conveniently shift the output signal in time and to generate a sigmoidal reaction kinetics. The obtained lag times can be freely tuned by simply varying the blocker concentration and the enzymatic degradation rate.

The simplicity of the strategy was key for the establishment of three different systems that can be operated in parallel as well as for the coupling to downstream reactions. Given the full orthogonality of the systems, it should be possible to sequentially cascade these systems to further sharpen the onsets/abruptness of the final output generation and extend the range of possible delays. Given that the blocker strands bind sequence-specifically to their toeholds, orthogonal timers could even be established using just a single enzyme. Given such a high degree of modularity, this will allow us to design a large variety of different orthogonal reactions that can work in the same solution without any significant cross-reactivity. Implementing our approach in DNA-based reaction networks and other temporally controlled logic circuits^64^ can find applications in the programmable hierarchical assembly of different DNA structures in the same batch and in the temporal control of drug-release and therapeutic DNA nanodevices.

## Experimental Section

### Chemicals

All reagent-grade chemicals, including DEPC-treated water, MgCl_2_, trizma hydrochloride, EDTA, NaCl, and 1,4-dithiothreitol (DTT), were purchased from Sigma-Aldrich (Italy) and used without further purifications. Bovine serum albumin was purchased from New England Biolabs (Beverly, MA, USA).

### Enzymes

UDG, Fpg, and RNase H recombinant were purchased from New England Biolabs (Beverly, MA, USA). Before use, RNase H was previously activated by incubation for 1 h at 37 °C in 50 mM Tris–HCl, 50 mM KCl, 3 mM MgCl_2_ in the presence of 50 mM DTT at pH 8.0. Human α-thrombin was purchased from Haematologic Technologies (USA). Fibrinogen from human plasma was provided by Merck (Germany).

### Oligonucleotides

Oligonucleotides employed in this work were synthesized, labeled, and HPLC-purified by Metabion International AG (Planegg, Germany) and used without further purification. The DNA oligonucleotides were dissolved in phosphate buffer 50 mM, pH 7.0, and stored at −20 °C until use. The RNA oligonucleotides were dissolved in DEPC-treated water and stored at −20 °C until use. All the sequences of the different systems are reported in the Supporting Information document.

### Fluorescence Experiments

Fluorescence kinetic measurements were carried out on a Tecan F200pro plate reader using the top reading mode with black, flat bottom nonbinding 96-well plates and a 100 μL final volume. The concentrations employed and buffer conditions are reported in the legend of each figure. Detailed procedures employed in the different experiments are reported in the Supporting Information document.

## Supporting Information Available

Additional experimental details and oligonucleotide sequences used; fluorescence emission spectra; native PAGE electrophoresis experiments; and control experiments, kinetic model, and curve fitting (PDF)

## References

[ref1] Seeman N. C.; Sleiman H. F. DNA Nanotechnology. Nat. Rev. Mater. 2017, 3, 17068 10.1038/natrevmats.2017.68.

[ref2] Krishnan Y.; Bathe M. Designer Nucleic Acids to Probe and Program the Cell. Trends Cell Biol. 2012, 22, 624–633. 10.1016/j.tcb.2012.10.001. 23140833

[ref3] Shen H.; Wang Y.; Wang J.; Li Z.; Yuan Q. Emerging Biomimetic Applications of DNA Nanotechnology. ACS Appl. Mater. Interfaces 2019, 11, 13859–13873. 10.1021/acsami.8b06175. 29939004

[ref4] Ma W.; Zhan Y.; Zhang Y.; Mao C.; Xie X.; Lin Y. The Biological Applications of DNA Nanomaterials: Current Challenges and Future Directions. Signal Transduction Targeted Ther. 2021, 6, 351 10.1038/s41392-021-00727-9. PMC8497566 34620843

[ref5] Li J.; Fan C. A DNA Nanodevice Boosts Tumour Immunity. Nat. Nanotechnol. 2021, 16, 1306–1307. 10.1038/s41565-021-01002-2. 34799685

[ref6] Lin M.; Wang J.; Zhou G.; Wang J.; Wu N.; Lu J.; Gao J.; Chen X.; Shi J.; Zuo X.; Fan C. Programmable Engineering of a Biosensing Interface with Tetrahedral DNA Nanostructures for Ultrasensitive DNA Detection. Angew. Chem., Int. Ed. 2015, 54, 2151–2155. 10.1002/anie.201410720. 25556850

[ref7] Jiang S.; Ge Z.; Mou S.; Yan H.; Fan C. Designer DNA Nanostructures for Therapeutics. Chem 2021, 7, 1156–1179. 10.1016/j.chempr.2020.10.025.

[ref8] Ranallo S.; Prévost-Tremblay C.; Idili A.; Vallée-Bélisle A.; Ricci F. Antibody-Powered Nucleic Acid Release Using a DNA-Based Nanomachine. Nat. Commun. 2017, 8, 15150 10.1038/ncomms15150. 28480878 PMC5424144

[ref9] Idili A.; Vallée-Bélisle A.; Ricci F. Programmable PH-Triggered DNA Nanoswitches. J. Am. Chem. Soc. 2014, 136, 5836–5839. 10.1021/ja500619w. 24716858

[ref10] Haydell M. W.; Centola M.; Adam V.; Valero J.; Famulok M. Temporal and Reversible Control of a DNAzyme by Orthogonal Photoswitching. J. Am. Chem. Soc. 2018, 140, 16868–16872. 10.1021/jacs.8b08738. 30444607

[ref11] Hu Y.; Cecconello A.; Idili A.; Ricci F.; Willner I. Triplex DNA Nanostructures: From Basic Properties to Applications. Angew. Chem., Int. Ed. 2017, 56, 15210–15233. 10.1002/anie.201701868. 28444822

[ref12] Zhang P.; Ouyang Y.; Sohn Y. S.; Nechushtai R.; Pikarsky E.; Fan C.; Willner I. PH- And MiRNA-Responsive DNA-Tetrahedra/Metal-Organic Framework Conjugates: Functional Sense-and-Treat Carriers. ACS Nano 2021, 15, 6645–6657. 10.1021/acsnano.0c09996. 33787219

[ref13] Centola M.; Valero J.; Famulok M. Allosteric Control of Oxidative Catalysis by a DNA Rotaxane Nanostructure. J. Am. Chem. Soc. 2017, 139, 16044–16047. 10.1021/jacs.7b08839. 29058418

[ref14] Wickham S. F. J.; Bath J.; Katsuda Y.; Endo M.; Hidaka K.; Sugiyama H.; Turberfield A. J. A DNA-Based Molecular Motor That Can Navigate a Network of Tracks. Nat. Nanotechnol. 2012, 7, 169–173. 10.1038/nnano.2011.253. 22266636

[ref15] Jester S. S.; Famulok M. Mechanically Interlocked DNA Nanostructures for Functional Devices. Acc. Chem. Res. 2014, 47, 1700–1709. 10.1021/ar400321h. 24627986

[ref16] Zhang D. Y.; Seelig G. Dynamic DNA Nanotechnology Using Strand-Displacement Reactions. Nat. Chem. 2011, 3, 103–113. 10.1038/nchem.957. 21258382

[ref17] Kahn J. S.; Hu Y.; Willner I. Stimuli-Responsive DNA-Based Hydrogels: From Basic Principles to Applications. Acc. Chem. Res. 2017, 50, 680–690. 10.1021/acs.accounts.6b00542. 28248486

[ref18] Wojciechowski J. P.; Martin A. D.; Thordarson P. Kinetically Controlled Lifetimes in Redox-Responsive Transient Supramolecular Hydrogels. J. Am. Chem. Soc. 2018, 140, 2869–2874. 10.1021/jacs.7b12198. 29406709

[ref19] Whitesides G. M.; Grzybowski B. Self-Assembly at All Scales. Science 2002, 295, 2418–2421. 10.1126/science.1070821. 11923529

[ref20] Dhiman S.; Jain A.; Kumar M.; George S. J. Adenosine-Phosphate-Fueled, Temporally Programmed Supramolecular Polymers with Multiple Transient States. J. Am. Chem. Soc. 2017, 139, 16568–16575. 10.1021/jacs.7b07469. 28845662

[ref21] Boekhoven J.; Hendriksen W. E.; Koper G. J. M.; Eelkema R.; van Esch J. H. Transient Assembly of Active Materials Fueled by a Chemical Reaction. Science 2015, 349, 1075–1079. 10.1126/science.aac6103. 26339025

[ref22] Merindol R.; Walther A. Materials Learning from Life: Concepts for Active, Adaptive and Autonomous Molecular Systems. Chem. Soc. Rev. 2017, 46, 5588–5619. 10.1039/c6cs00738d. 28134366

[ref23] Prigogine I.; Lefever R.; Goldbeter A.; Herschkowitz-kaufman M. Symmetry Breaking Instabilities in Biological Systems. Nature 1969, 223, 913–916. 10.1038/223913a0. 5803393

[ref24] Xiong W.; Ferrell J. E. A positive-feedback-based bistable ’memory module’ that governs a cell fate decision. Nature 2003, 426, 460–465. 10.1038/nature02089. 14647386

[ref25] Semenov S. N.; Kraft L. J.; Ainla A.; Zhao M.; Baghbanzadeh M.; Campbell V. E.; Kang K.; Fox J. M.; Whitesides G. M. Autocatalytic, Bistable, Oscillatory Networks of Biologically Relevant Organic Reactions. Nature 2016, 537, 656–660. 10.1038/nature19776. 27680939

[ref26] Gérard C.; Goldbeter A. Temporal Self-Organization of the Cyclin/Cdk Network Driving the Mammalian Cell Cycle. Proc. Natl. Acad. Sci. U.S.A. 2009, 106, 21643–21648. 10.1073/pnas.090382710. 20007375 PMC2799800

[ref27] Machinek R. R. F.; Ouldridge T. E.; Haley N. E. C.; Bath J.; Turberfield A. J. Programmable Energy Landscapes for Kinetic Control of DNA Strand Displacement. Nat. Commun. 2014, 5, 5324 10.1038/ncomms6324. 25382214

[ref28] Srinivas N.; Ouldridge T. E.; Šulc P.; Schaeffer J. M.; Yurke B.; Louis A. A.; Doye J. P. K.; Winfree E. On the Biophysics and Kinetics of Toehold-Mediated DNA Strand Displacement. Nucleic Acids Res. 2013, 41, 10641–10658. 10.1093/nar/gkt801. 24019238 PMC3905871

[ref29] Irmisch P.; Ouldridge T. E.; Seidel R. Modeling DNA-Strand Displacement Reactions in the Presence of Base-Pair Mismatches. J. Am. Chem. Soc. 2020, 142, 11451–11463. 10.1021/jacs.0c03105. 32496760

[ref30] Haley N. E. C.; Ouldridge T. E.; Mullor Ruiz I.; Geraldini A.; Louis A. A.; Bath J.; Turberfield A. J. Design of Hidden Thermodynamic Driving for Non-Equilibrium Systems via Mismatch Elimination during DNA Strand Displacement. Nat. Commun. 2020, 11, 2562 10.1038/s41467-020-16353-y. 32444600 PMC7244503

[ref31] Foo M.; Sawlekar R.; Kulkarni V. v.; Bates D. G. Biologically Inspired Design of Feedback Control Systems Implemented Using DNA Strand Displacement Reactions. Annual International Conference of the IEEE Engineering in Medicine and Biology Society; IEEE, 2016; pp 1455–1458. 10.1109/EMBC.2016.7590983 28268600

[ref32] Joesaar A.; Yang S.; Bögels B.; van der Linden A.; Pieters P.; Kumar B. V. V. S. P.; Dalchau N.; Phillips A.; Mann S.; de Greef T. F. A. DNA-Based Communication in Populations of Synthetic Protocells. Nat. Nanotechnol. 2019, 14, 369–378. 10.1038/s41565-019-0399-9. 30833694 PMC6451639

[ref33] Heinen L.; Walther A. Programmable Dynamic Steady States in ATP-Driven Nonequilibrium DNA Systems. Sci. Adv. 2019, 5, eaaw0590 10.1126/sciadv.aaw0590. 31334349 PMC6641946

[ref34] Deng J.; Walther A. Pathway Complexity in Fuel-Driven DNA Nanostructures with Autonomous Reconfiguration of Multiple Dynamic Steady States. J. Am. Chem. Soc. 2020, 142, 685–689. 10.1021/jacs.9b11598. 31895547 PMC7612462

[ref35] Del Grosso E.; Amodio A.; Ragazzon G.; Prins L.; Ricci F. Dissipative Synthetic DNA-Based Receptors for the Transient Loading and Release of Molecular Cargo. Angew. Chem., Int. Ed. 2018, 130, 10649–10653. 10.1002/ange.201801318. 29603570

[ref36] Del Grosso E.; Irmisch P.; Gentile S.; Prins L. J.; Seidel R.; Ricci F. Dissipative Control over the Toehold-Mediated DNA Strand Displacement Reaction. Angew. Chem., Int. Ed. 2022, 61, e202201929 10.1002/anie.202201929. PMC9324813 35315568

[ref37] Wang J.; Li Z.; Zhou Z.; Ouyang Y.; Zhang J.; Ma X.; Tian H.; Willner I. DNAzyme- and Light-Induced Dissipative and Gated DNA Networks. Chem. Sci. 2021, 12, 11204–11212. 10.1039/d1sc02091a. 34522318 PMC8386649

[ref38] Li Z.; Wang J.; Willner I. Transient Out-of-Equilibrium Nucleic Acid-Based Dissipative Networks and Their Applications. Adv. Funct. Mater. 2022, 32, 2200799 10.1002/adfm.202200799.

[ref39] Kim J.; Winfree E. Synthetic in Vitro Transcriptional Oscillators. Mol. Syst. Biol. 2011, 7, 465 10.1038/msb.2010.119. 21283141 PMC3063688

[ref40] Franco E.; Friedrichs E.; Kim J.; Jungmann R.; Murray R.; Winfree E.; Simmel F. C. Timing Molecular Motion and Production with a Synthetic Transcriptional Clock. Proc. Natl. Acad. Sci. U.S.A. 2011, 108, E784–E793. 10.1073/pnas.1100060108. 21921236 PMC3189071

[ref41] Weitz M.; Kim J.; Kapsner K.; Winfree E.; Franco E.; Simmel F. C. Diversity in the Dynamical Behaviour of a Compartmentalized Programmable Biochemical Oscillator. Nat. Chem. 2014, 6, 295–302. 10.1038/nchem.1869. 24651195

[ref42] Montagne K.; Plasson R.; Sakai Y.; Fujii T.; Rondelez Y. Programming an in Vitro DNA Oscillator Using a Molecular Networking Strategy. Mol. Syst. Biol. 2011, 7, 476 10.1038/msb.2011.12. PMC3063689 21283142

[ref43] Gentile S.; Del Grosso E.; Pungchai P. E.; Franco E.; Prins L. J.; Ricci F. Spontaneous Reorganization of DNA-Based Polymers in Higher Ordered Structures Fueled by RNA. J. Am. Chem. Soc. 2021, 143, 20296–20301. 10.1021/jacs.1c09503. 34843256 PMC8662731

[ref44] Zhou Z.; Ouyang Y.; Wang J.; Willner I. Dissipative Gated and Cascaded DNA Networks. J. Am. Chem. Soc. 2021, 143, 5071–5079. 10.1021/jacs.1c00486. 33755445

[ref45] Del Grosso E.; Amodio A.; Ragazzon G.; Prins L. J.; Ricci F. Dissipative Synthetic DNA-Based Receptors for the Transient Loading and Release of Molecular Cargo. Angew. Chem., Int. Ed. 2018, 57, 10489–10493. 10.1002/anie.201801318. 29603570

[ref46] Del Grosso E.; Franco E.; Prins L. J.; Ricci L. J. F. Dissipative DNA nanotechnology. Nat. Chem. 2022, 14, 600–613. 10.1038/s41557-022-00957-6. 35668213

[ref47] Fern J.; Scalise D.; Cangialosi A.; Howie D.; Potters L.; Schulman R. DNA Strand-Displacement Timer Circuits. ACS Synth. Biol. 2017, 6, 190–193. 10.1021/acssynbio.6b00170. 27744682

[ref48] Scalise D.; Rubanov M.; Miller K.; Potters L.; Noble M.; Schulman R. Programming the Sequential Release of DNA. ACS Synth. Biol. 2020, 9, 749–755. 10.1021/acssynbio.9b00398. 32212717

[ref49] Srinivas N.; Parkin J.; Seelig G.; Winfree E.; Soloveichik D. Enzyme-Free Nucleic Acid Dynamical Systems. Science 2017, 358, eaal2052 10.1126/science.aal2052. 29242317

[ref50] Deng J.; Walther A. ATP-Responsive and ATP-Fueled Self-Assembling Systems and Materials. Adv. Mater. 2020, 32, 2002629 10.1002/adma.202002629. 32881127

[ref51] Yurke B.; Turberfield A. J.; Mills A. P.; Simmel F. C.; Neumann J. L. A DNA-Fuelled Molecular Machine Made of DNA. Nature 2000, 406, 605–608. 10.1038/35020524. 10949296

[ref52] Zhang D. Y.; Winfree E. Control of DNA Strand Displacement Kinetics Using Toehold Exchange. J. Am. Chem. Soc. 2009, 131, 17303–17314. 10.1021/ja906987s. 19894722

[ref53] Zhang D. Y.; Hariadi R. F.; Choi H. M. T.; Winfree E. Integrating DNA Strand-Displacement Circuitry with DNA Tile Self-Assembly. Nat. Commun. 2013, 4, 1965 10.1038/ncomms2965. 23756381 PMC3709499

[ref54] Simmel F. C.; Yurke B.; Singh H. R. Principles and Applications of Nucleic Acid Strand Displacement Reactions. Chem. Rev. 2019, 119, 6326–6369. 10.1021/acs.chemrev.8b00580. 30714375

[ref55] Liu M.; Fu J.; Hejesen C.; Yang Y.; Woodbury N. W.; Gothelf K.; Liu Y.; Yan H. A DNA Tweezer-Actuated Enzyme Nanoreactor. Nat. Commun. 2013, 4, 2127 10.1038/ncomms3127. 23820332

[ref56] Green L. N.; Subramanian H. K. K.; Mardanlou V.; Kim J.; Hariadi R. F.; Franco E. Autonomous Dynamic Control of DNA Nanostructure Self-Assembly. Nat. Chem. 2019, 11, 510–520. 10.1038/s41557-019-0251-8. 31011170

[ref57] Cerritelli S. M.; Crouch R. J. H. Ribonuclease H: the enzymes in eukaryotes. FEBS J. 2009, 276, 1494–1505. 10.1111/j.1742-4658.2009.06908.x. 19228196 PMC2746905

[ref58] Bruner S. D.; Norman D. P. G.; Verdine G. L. Structural Basis for Recognition and Repair of the Endogenous Mutagen 8-Oxoguanine in DNA. Nature 2000, 403, 859–866. 10.1038/35002510. 10706276

[ref59] Schormann N.; Ricciardi R.; Chattopadhyay D. Uracil-DNA Glycosylases-Structural and Functional Perspectives on an Essential Family of DNA Repair Enzymes. Protein Sci. 2014, 23, 1667–1685. 10.1002/pro.2554. 25252105 PMC4253808

[ref60] Del Grosso E.; Idili A.; Porchetta A.; Ricci F. A Modular Clamp-like Mechanism to Regulate the Activity of Nucleic-Acid Target-Responsive Nanoswitches with External Activators. Nanoscale 2016, 8, 18057–18061. 10.1039/c6nr06026a. 27714163

[ref61] Bock L. C.; Griffin L. C.; Latham J. A.; Vermaas E. H.; Toole J. J. Selection of Single-Stranded DNA Molecules That Bind and Inhibit Human Thrombin. Nature 1992, 355, 564–566. 10.1038/355564a0. 1741036

[ref62] Wang F.; Lv H.; Li Q.; Li J.; Zhang X.; Shi J.; Wang L.; Fan C. Implementing Digital Computing with DNA-Based Switching Circuits. Nat. Commun. 2020, 11, 121 10.1038/s41467-019-13980-y. 31913309 PMC6949259

[ref63] Deng J.; Walther A. Fuel-Driven Transient DNA Strand Displacement Circuitry with Self-Resetting Function. J. Am. Chem. Soc. 2020, 142, 21102–21109. 10.1021/jacs.0c09681. 33322910 PMC7612460

[ref64] Lapteva A.; Sarraf N.; Qian L. DNA Strand-Displacement Temporal Logic Circuits. J. Am. Chem. Soc. 2022, 144, 12443–12449. 10.1021/jacs.2c04325. 35785961 PMC9284558

