# Supplementary material for: Orthogonal Enzyme-Driven Timers for DNA Strand Displacement Reactions
Source: J Am Chem Soc. 2022 Oct 18;144(43):19791–8. doi: 10.1021/jacs.2c06599 (PMC9634797; doi:10.1021/jacs.2c06599)
Supplement: Supplementary file 1 — ja2c06599_si_001.pdf [file ja2c06599_si_001.pdf]

Supporting Information

Orthogonal Enzyme-Driven Timers for DNA Strand Displacement Reactions

Juliette Bucci,[1] Patrick Irmisch,[2] Erica Del Grosso,[1] Ralf Seidel,[2] and Francesco Ricci,[1,*]

[1]Chemistry Department, University of Rome, Tor Vergata, Via della Ricerca Scientifica, 00133, Rome, Italy.
[2] Molecular Biophysics Group, Peter Debye Institute for Soft Matter Physics, Universität Leipzig, 04103 Leipzig, Germany;

Table of contents

1.Experimental Procedures S3

Oligonucleotides S3

Fluorescence experiments S6

Kinetic measurements S6

Emission spectra measurements S7

Native-PAGE gel-imaging S7

Light scattering experiments S8

2.Kinetic modelling and curve fitting S8

a. General considerations S8

b. General rate model for DNA strand displacement reactions with enzyme-based delays S9

c. Curve fitting S10

d. Rate model for the RNase H-based strand displacement reactions with delay S10

e. Rate model for the Fpg-based strand displacement reactions with delay S11

f. Rate model for the UDG-based strand displacement reactions with delay S12

g. Rate model for the UDG-based delay of DNA cargo release from the DNA receptor S12

3.Supplementary figures S14

References S32

1.Experimental Procedures

Oligonucleotides

Sequences for RNase H, UDG and Fpg controlled strand displacement systems are listed below.

System 1: RNase H-based DNA strand displacement reaction with delay

**Name**	**Sequence**
Target	5'- ATC T**TC ATA CAC GTT GCT AG**G TCT CGC TAT CAG GAT CTA T- BHQ-2 -3'
Output	5'- Cy3 - ATA GAT CCT GAT AGC GAG AC -3'
RNA-blocker	5'- CUA GCA ACG UGU AUG A -3'
Input	5'- ATA GAT CCT GAT AGC GAG ACC TAG C -3'

The output and target sequences represent the two complementary strands of the target duplex, that is conjugated to a fluorophore (Cy3) and a quencher (BHQ-2). The bold bases in the target strand represent the blocker-binding region. The underlined bases in the target, RNA-blocker and input strand represent the toehold and toehold-binding domains.

System 2: UDG-based DNA strand displacement reaction with delay

**Name**	**Sequence**
Target	5'- AAC A**CT TCA CAA CTA CAG CT**T CAA TTC AGG ACA ATC GGC T- BHQ-2 -3'
Output	5'- Cy5 - AGC CGA TTG TCC TGA ATT GA -3'
Uracil-blocker	5'- AGC (2- Deoxyuridine)G(2- Deoxyuridine) AG(2- Deoxyuridine) TG(2- Deoxyuridine) GAA G -3'
Input	5'- AGC CGA TTG TCC TGA ATT GAA GCT G -3'

The output and target sequences represent the two complementary strands of the target duplex, that are conjugated to a fluorophore (Cy5) and a quencher (BHQ-2). The bold bases in the target strand represent the complementary blocker portion. The underlined bases in the target, uracil-blocker and input strand represent the toehold and toehold-binding domains.

System 3: Fpg-based DNA strand displacement reaction with delay

Name	Sequence
Target	5'- ATT C**CA AAC CAC ATT ACC CA**A CAA ACA GAC GCA ATC CAC T - BHQ-1 -3'
Output	5'- 6-Fam - AGT GGA TTG CGT CTG TTT GT -3'
G^{OXO}-blocker	5'- TGG GTA AT(8-oxo-dG) TGG TTT G -3'
Input	5'- AGT GGA TTG CGT CTG TTT GTT GGG T -3'

The output and target sequences represent the two complementary strands of the target duplex, that are conjugated to a fluorophore (6-FAM) and a quencher (BHQ-1). The bold bases in the target strand represent the complementary blocker-portion. The underlined bases in the target, G^{oxo}-blocker and input strand represent toehold and toehold-binding domains.

System 4: UDG-based delay of DNA cargo release from a DNA receptor

Name	Sequence
Target	5'- ATC T**TC ATA CAC GTT GCT AG**T CAC TGG TCC CTT TGA ATG C -3'
Output	5'- **GAA GAA AAG CAT TCA AAG** GGA CCA GTG A -3'
Uracil-blocker	5'- C(2-Deoxyuridine)A GCA ACG (2-Deoxyuridine)G(2-Deoxyuridine) A(2-Deoxyuridine)GA -3'
Input	5'- TTC AAA GGG ACC AGT GAC TAG C -3'
Ligand-binding device	5'- *TTC CCT CTT CTT CCT* **CCT TTT AAT GAT TTT CAT** *CC TTC TTC TCC CTT* -3'
Ligand	5'- 6-Fam - AGA AAG GAG -BHQ2 -3'

The output and target sequences represent the two complementary strands of the target duplex. The bold bases in the output strand represent the complementary portion to the receptor. The bold bases in the target strand represent the complementary portion to the uracil-blocker. The underlined bases in the target, uracil-blocker and input strand represent the toehold and toehold-binding domains.

For the ligand-binding device sequence the bold bases represent the portion complementary to the output, the underlined bases represent the ligand-binding site (through Watson-Crick bonds) and italics bases represent the triplex-forming portion.

System 5: RNase H-based temporal-controlled of thrombin activity

Name	Sequence
Target	5'- CCA ACC ACA CCA ACC **TCT CTC CTT TCT CTG A**TA CT -3'
Output	5'- GGT TGG TGT GGT TGG -3'
RNA-blocker	5'- UCA GAG AAA GGA GAG A -3'
Input	5'- AGA GAG GTT GGT GTG -3'

The output (thrombin-binding aptamer) and target sequences represent the two complementary strands of the target duplex. The bold bases in the target strand represent the complementary portion to the RNA-blocker. The underlined bases in the target, RNA-blocker and input strand represent the toehold and toehold-binding domains.

System 6: Enzyme-based temporally controlled DNA logic circuit

Name	Sequence
Target 1	5'- BHQ-2- TAT CAT GGT GTA TCG GAG TGC A**GC TTA CCA CAT ACT** -3'
Output 1	5'- CAC TCC GAT ACA CCA TGA TA-Cy3 -3'
RNA-blocker	5'- AGU AUG UGG UAA GCU G -3'
Target 2	5'- **CT TCA CAA CTA CAG CT**T CAA TTC AGG ACA ATC GGC T-BHQ-2 -3'
Output 2	5'- Cy5- AGC CGA TTG TCC TGA ATT GA -3'
Uracil-blocker	5'- AGC (2- Deoxyuridine)G(2- Deoxyuridine) AG(2- Deoxyuridine) TG(2- Deoxyuridine) GAA G -3'
Input	5'- AGC CGA TTG TCC TGA ATT GA AGCTG CAC TCC GAT ACA CCA TGA TA -3'

The output and target sequences represent the two complementary strands of the respective target duplex. The bold bases in the target strands represent the complementary portion to the blocker. The underlined bases in the targets, RNA-blocker, uracil-blocker and input strand represent the toehold and toehold-binding domains, respectively.

Fluorescence experiments

Kinetic measurements

Fluorescence kinetic measurements were carried out on a Tecan F200pro plate reader using the top reading mode with black, flat bottom non-binding 96-well plates. The working wavelengths were set to λ_{exc} = 540 (±25) nm and λ_{emi} = 595 (±35) nm for the Cy3 labeled oligonucleotides, λ_{exc} = 644 (±10) nm and λ_{emi}= 670 (±10) nm for the Cy5 labeled oligonucleotides and λ_{exc} = 485 (±20) nm and λ_{emi} = 535 (±25) nm for the 6-Fam labeled oligonucleotides.

Enzyme-controlled delayed strand displacement reactions (RNase H, UDG and Fpg)

All experiments shown in Figures 2 and 3, were performed at 30°C in 20 mM Tris-HCl buffer, 10 mM $MgCl_2$, 1 mM EDTA, pH 8.0. The initial target duplex (50 nM) was formed by incubating at 90°C for 2 minutes target and output strands in presence of the desired concentration of blocker strands. Only for the system with the RNA-blocker the incubation was performed at 60°C. After 30 minutes the preformed duplex with blocker was transferred (100 μL) to a 96-well plates where 10 mM DTT, 0.1 mg/mL BSA and 50 nM input strand were added. After the stabilization of the signal, the corresponding enzyme was added in the wells at the desired concentration and the fluorescence intensity was recorded over time.

Orthogonal temporal control of strand displacement reactions

All experiments shown in Figure 4 were performed at 30°C in 20 mM Tris-HCl buffer, 10 mM $MgCl_2$, 1 mM EDTA, pH 8.0. The target duplex for each enzyme-controlled strand displacement system were incubated together at 60°C for 2 minutes, using equimolar concentrations of the two relevant strands (target and output) and the desired concentration of blockers. After 30 minutes the preformed duplexes were transferred (100 μL) to 96-well plates where DTT (10 mM) BSA (0.1 mg/mL) and the three input strands (50 nM) were added. After the stabilization of the signal, the three enzymes (at the desired concentration) were added into the wells at the same time and the fluorescence intensity was recorded over time.

UDG-based delay of DNA ligand release from a DNA device

All experiments shown in Figure 5 were performed at 25°C in 10 mM Tris-HCl buffer, 3 mM $MgCl_2$, pH 6.0. The target duplex was prepared as previously described and then transferred (100 μL) to a 96-well plates, where the ligand-binding device (50 nM), the ligand (50 nM) and

the input (50 nM) were added. After the stabilization of the signal, the enzyme (at the desired concentration) was added into the well.

Enzyme-based temporally controlled DNA logic circuit

All experiments shown in Figure 7 were performed at 30°C in 20 mM Tris-HCl buffer, 10 mM $MgCl_2$, 1 mM EDTA, pH 8.0. The target duplexes for each enzyme-controlled strand displacement system were incubated together at 60°C for 2 minutes, using equimolar concentrations of the two relevant strands (target and output) and the desired concentration of blockers (150 nM). After 30 minutes the preformed duplexes were transferred (100 μL) to a 96-well plate where DTT (10 mM) and the input strand (100 nM) were added. After the stabilization of the signal, the two enzymes (at the desired concentration) were added into the wells at the same time and the fluorescence intensity for the two outputs was recorded over time.

Emission spectra measurements

Fluorescence spectra measurements were carried out on a Cary Eclipse Fluorimeter (Varian). The working wavelengths were as follows: λ_{ex} = 543 nm ($slit_{ex}$ = 5 nm) and acquisition between 555 and 700 nm ($slit_{em}$ = 10 nm) for the Cy3 labeled oligonucleotides; λ_{ex} = 640 nm ($slit_{ex}$ = 5 nm) and acquisition between 655 and 800 nm ($slit_{em}$ = 10 nm) for the Cy5 labeled oligonucleotides; λ_{ex} = 490 nm ($slit_{ex}$ = 5 nm) and acquisition between 506 and 650 nm ($slit_{em}$ = 10 nm) for the 6-Fam labeled oligonucleotides. All measurements were performed using quartz cuvettes (100 µL) at T = 30°C in 20 mM Tris-HCl buffer, 10 mM $MgCl_2$, 1 mM EDTA, pH 8.0.

Native-PAGE gel-imaging

All experiments shown in Figures S2,S7 and S10 were performed at 30°C in 20 mM Tris-HCl buffer, 10 mM $MgCl_2$, 1 mM EDTA, pH 8.0. The initial target duplexes (50 nM) were formed using the procedure previously described. After 30 minutes the input strand (50 nM) was added to the preformed complex and the solution was incubated at 30°C for 15 minutes. Enzyme (at the desired concentration) was then added into the reaction mixture. The reactions were stopped at different times by incubation at 4°C. Samples were diluted to 30 nM in a buffer solution containing 60% glycerol and directly loaded on 15% acrylamide native-PAGE. The gel was allowed to run for 120 minutes at 120 V. The bands were detected by direct gel-imaging using the ChemiDocTM MP imaging system (Bio-Rad).

Light scattering experiments

RNase H temporally-controlled thrombin activity

The target duplex (50 nM) was formed by incubating at 60°C for 2 minutes at equimolar concentrations of the two relevant strands (target and output) with the desired concentration of blocker in 20 mM Tris-HCl buffer, 10 mM $MgCl_2$, 1 mM EDTA, 150 mM NaCl, pH 8.0. The input strand (50 nM) was then added to the preformed complex and the solution was incubated at 30°C for 15 minutes. RNase H (at the desired concentration) was then added into the reaction mixture. Aliquots of 117 µL were withdrawn at different times from the above reaction mixture and mixed with 13 µL of fibrinogen (1mg/mL) and then transferred into a quartz cuvette. After the addition of thrombin (0.5 nM) the time-dependent light scattering changes due to the formation and aggregation of insoluble fibrin resulting from thrombin enzymatic activity were monitored at 350 nm at 25°C using a Varian Cary 100 UV-Vis spectrophotometer.

2. Kinetic modelling and curve fitting

a. General considerations

To model the experimental results obtained in this work, we developed a minimalistic reaction schemes that included only kinetically-relevant reaction steps by neglecting very fast forward reactions and slow reverse reactions.[1] In particular, strand displacement reactions were described as single-step, irreversible reactions and for enzymatic reactions, protein activity is always assumed to happen under saturation conditions (i.e. concentrations of protein and substrates well above the K_D). All considered reaction steps were assumed to be of first or second order. Numeric integration of the sets of differential equations resulting from the reaction schemes provided the time courses of the concentrations of the different reaction species.

The conventional strand displacement reactions (Figure S4) were assumed to follow simple second order kinetics with the displacement rate constant k_{displ}:

$$AB + C \xrightarrow{k_{\mathrm{displ.}}} AC + B, \tag{1}$$

where the target strand (*A*) and the output strand (*B*) form an initial target duplex (*AB*), which reacts with an input strand (*C*) to release the output. The set of rate equations, that describe strand displacement are:

$$\frac{d[AB]}{dt} = \frac{d[C]}{dt} = \quad - k_{\text{displ.}}[AB][C], \tag{2}$$

$$\frac{d[AC]}{dt} = \frac{d[B]}{dt} = \quad + k_{\text{displ.}}[AB][C]. \tag{3}$$

The obtained time-course of the output concentration was related to the measured fluorescence signal F using a linear scaling factor *S*:

$$F = S[B]. \tag{4}$$

b. General rate model for DNA strand displacement reactions with enzyme-based delays

For the delayed strand displacement reactions, first a general/complete reaction scheme is developed that is applicable to the three different enzyme systems (Figure S5). Adaptation of the model to the particular enzyme system allowed partial exclusion of certain reaction steps.

In the starting configuration, the blocker strands (*O*) are bound to the toehold region of the initial target duplex (*AB*) to form the complex *ABO*. Degradation of the blocker strands on the output-target complex is assumed to occur under pseudo-first order conditions with rate constant k_{deg} such that it is described by the reaction:

$$ABO \xrightarrow{k_{\text{deg}}} AB + R. \tag{5}$$

during which unblocked output-target complexes *AB* as well as degraded blocker fragments *R* are produced. After unblocking, the target duplex can either rebind another blocker strand with the rate constant k_{bind} (as long as intact blockers are in solution):

$$AB + O \xrightarrow{k_{\text{bind}}} ABO, \tag{6}$$

or react with the input strand *C* to release the output strand *B* by strand displacement:

$$AB + C \xrightarrow{k_{\text{displ.}}} AC + B, \tag{7}$$

Furthermore, we allow the parallel degradation of unbound (free) blocker strands in solution with the rate constant $\gamma\, k_{\text{deg}}$:

$$O \xrightarrow{\gamma\, k_{\text{deg}}} R. \tag{8}$$

where γ is a dimensionless scaling factor for the degradation rate constant of bound blocker k_{deg}. Given pseudo-first order conditions, k_{deg} is generally assumed to be proportional to the enzyme concentration [*E*] but can become reduced by enzyme-inhibition due to accumulation of degraded fragments *R*. For convenience we took the enzyme concentration

given in U/ml as a dimensionless scaling factor for k_{deg}. Using the Michaelis-Menten scheme for competitive inhibition allows to express the degradation rate as:

$$k_{\mathrm{deg}} = \frac{k_{\mathrm{E}}[E]}{1 + \frac{[R]}{K_{\mathrm{R}}}}, \tag{9}$$

where k_{E} is the degradation rate at 1 U/ml and K_{R} is the dissociation constant of the degraded fragments from the enzyme.

The set of differential equations describing the general

$$\frac{d[ABO]}{dt} = -k_{\mathrm{deg}}[ABO] + k_{\mathrm{bind}}[AB][O] \tag{10}$$

$$\frac{d[AB]}{dt} = +k_{\mathrm{deg}}[ABO] - k_{\mathrm{bind}}[AB][O] - k_{\mathrm{displ}}[AB][C], \tag{11}$$

$$\frac{d[O]}{dt} = -k_{\mathrm{bind}}[AB][O] - \gamma k_{\mathrm{deg}}[O], \tag{12}$$

$$\frac{d[C]}{dt} = -k_{\mathrm{displ}}[AB][C], \tag{13}$$

$$\frac{d[AC]}{dt} = \frac{d[B]}{dt} = +k_{\mathrm{displ}}[AB][C], \tag{14}$$

$$\frac{d[R]}{dt} = +k_{\mathrm{deg}}[ABO] + \gamma k_{\mathrm{deg}}[O]. \tag{15}$$

The obtained time course for the output strands (B) can again be related to measured fluorescence signal using Equation 4.

c. Curve fitting

All curve fitting was performed using self-written Python (version 3.7) scripts. The scripts use the "odeint" function to numerically integrate a set of ordinary differential equations. The solution was then fitted to the measured kinetics using the nonlinear least-squares function "curve_fit". Both functions are part of the SciPy package.[2]

d. Rate model for the RNase H-based strand displacement reactions with delay

For the RNase H-based reactions (data in Figure 2c,e) the general reaction scheme in Figure S5 could be simplified. The RNase H activity is highly specific on RNA-DNA hetero-duplexes such that free RNA-blocker degradation can be neglected for which $\gamma = 0$. This effectively eliminates the second terms on the right side in Equations 12 and 15.

Additionally, noticeable inhibition of the enzyme activity was not observed when modelling the data, i.e. consideration of an inhibition provided high values for the dissociation constant

K_R, such that the competitive inhibition correction in Equation 7 could be neglected, providing a simple linear scaling of the degradation with enzyme concentration ($k_{\text{deg}} = k_E[E]$).

When fitting the model to experimental data, we first obtained the displacement rate constant by performing a measurement in the absence of blocker strands (Figure S14a) and applying Equations 2-4. The displacement rate constant for the RNase H system was determined to be $k_{\text{displ}} = (1.15 \pm 0.02) \cdot 10^5/Ms$.

Then we performed global fits of the blocker and enzyme concentration dependent series (Figure 2c,e) to obtain the other rate constants of the differential equation set (k_{bind} and k_{E}). For the rebinding rate constant $k_{\text{bind}} = (7.0 \pm 0.3) \cdot 10^5/Ms$, a single value was taken for all traces. To account for small variations in enzyme concentration/activity, which are mainly caused by pipetting errors, the activity rate constant k_{E} was an individual fit parameter for each trace (Figure S15). Also individual values were taken for the fluorescence scaling factor S. To predict the reaction half-life times as function of blocker or enzyme concentration (Figure 2d,f), we used the average values of the activity rate constants for each set (Figure S15, $k_{\text{E1}} = (0.7 \pm 0.2) \cdot 10^{-4}/s$ and $k_{\text{E2}} = (1.2 \pm 0.1) \cdot 10^{-4}/s$).

e. Rate model for the Fpg-based strand displacement reactions with delay

For the Fpg-based reactions (data Figure 3b and Figure S6c,d), free blocker degradation can be neglected ($\gamma = 0$, see RNAse-H system above), because Fpg shows negligible activity on single stranded compared to double stranded DNA[3], Fpg inhibition by waste fragments was however considered, since obtained values for K_R were in the order of the blocker concentration.

The general fitting approach was the same as described for the RNase H system. We determined the displacement rate constant of the Fpg system $k_{\text{displ}} = (4.7 \pm 0.2) \cdot 10^5/Ms$ from fitting displacement experiments in the absence of blocker strands (Figure S14c) using equations 2-4.

From a global fit of the blocker and enzyme concentration dependent series (Figure S6c,d), we obtained for the rebinding rate constant $k_{\text{bind}} = (3.99 \pm 0.02) \cdot 10^6/Ms$, and for the dissociation constant of the degradation fragments $K_R = (86.1 \pm 0.5)\, nM$. Enzyme activity rate constants k_{E} were obtained individually for each trace (Figure S16). To predict the reaction half-life times as function of blocker or enzyme concentration (Figure 3c,d), we

applied the average value of the activity rate constants $k_{\mathrm{E}} = (5.7 \pm 2.1) \cdot 10^{-4}/s$, since the variation between the experiments were low, thus supporting our approach.

f. Rate model for the UDG-based strand displacement reactions with delay

For the UDG-based reactions (Figure 3f and Figure S9c,d), we considered free blocker degradation as well as enzyme inhibition by degradation fragments. UDG has a significantly higher activity on single stranded compared to double stranded DNA[4,5], such $\gamma > 0$.

The general fitting approach was the same as described for the other two enzymes. We determined the displacement rate constant for the UDG system $k_{\mathrm{displ}} = (1.56 \pm 0.03) \cdot 10^5/Ms$ from fitting displacement experiments in the absence of blocker strands (Figure S14b) and applying equations 2-4.

From a global fit of the blocker and enzyme concentration dependent series (Figure S9c,d) we obtained for the rebinding rate constant $k_{\mathrm{bind}} = (2.11 \pm 0.03) \cdot 10^6/Ms$, for the dissociation constant of the degradation fragments $K_R = (1.66 \pm 0.02)\ \mu M$ and the enzyme activity scaling factor on free blockers $\gamma = 4.1 \pm 0.1$. Enzyme activity rate constants k_{E} were obtained individually for each trace (Figure S17). To predict the reaction half-life times as function of blocker or enzyme concentration (Figure 3g,h), we applied the average values of the activity rate constants for each set ($k_{\mathrm{E1}} = (1.1 \pm 0.2) \cdot 10^{-3}/s$ and $k_{\mathrm{E2}} = (1.5 \pm 0.3) \cdot 10^{-3}/s$).

g. Rate model for the UDG-based delay of DNA cargo release from the DNA receptor

For modelling the delayed DNA cargo release from the DNA receptor (Figure 5), we employed the rate model for the UDG-based strand displacement reactions and extended it by an additional second order reaction for the ligand release. During this step, the output strand *B* binds to the cargo-loaded DNA-based nano-device *NG* and displaces the cargo *G* with rate constant k_{cargo}:

$$NG + B \xrightarrow{k_{\mathrm{cargo}}} NB + G \tag{16}$$

This modifies the previous set of differential equations to:

$$\frac{d[ABO]}{dt} = -k_{\mathrm{deg}}[ABO] + k_{\mathrm{rebind}}[AB][O], \tag{17}$$

$$\frac{d[AB]}{dt} = +k_{\text{deg}}[ABO] - k_{\text{rebind}}[AB][O] - k_{\text{displ}}[AB][C], \tag{18}$$

$$\frac{d[O]}{dt} = -k_{\text{rebind}}[AB][O] - \gamma k_{\text{deg}}[O], \tag{19}$$

$$\frac{d[C]}{dt} = -k_{\text{displ}}[AB][C], \tag{20}$$

$$\frac{d[AC]}{dt} = +k_{\text{displ}}[AB][C], \tag{21}$$

$$\frac{d[B]}{dt} = -k_{\text{cargo}}[B][NG], \tag{22}$$

$$\frac{d[R]}{dt} = +k_{\text{deg}}[ABO] + \gamma k_{\text{deg}}[O], \tag{23}$$

$$\frac{d[NG]}{dt} = -k_{\text{cargo}}[B][NG], \tag{24}$$

$$\frac{d[G]}{dt} = +k_{\text{cargo}}[B][NG], \tag{25}$$

$$\frac{d[NB]}{dt} = +k_{\text{cargo}}[B][NG]. \tag{26}$$

The measured fluorescence decrease was related to concentration of liberated cargo using a linear relation:

$$F = (S_0 - S_{inf})[NG] + S_{inf}, \tag{27}$$

where S_0 is the intensity of the fully bound cargo and S_{inf} the intensity after full release of the cargo, which were free parameters for each trace. Additionally, we included a small fraction of output strands $[B]_0$, which were already present at the start of the reaction due to small errors in pipetting the initial target duplex AB.

Since the reaction conditions for the application of the UDG system differed from that of the characterization (i.e. buffer composition, temperature and pH-value), we employed a new set of rate constants. From a global fit of the cargo release data (Figure 5b,d) we obtained the displacement rate constant $k_{\text{displ}} = (1.47 \pm 0.03) \cdot 10^5/Ms$, the rebinding rate constant $k_{\text{bind}} = (3.29 \pm 0.06) \cdot 10^5/Ms$, the dissociation constant of the degradation fragments $k_R = (1.06 \pm 0.02)\ \mu M$, the cargo release rate constant $k_{\text{cargo}} = (1.60 \pm 0.02) \cdot 10^4/Ms$, the scaling factor $\gamma = 0.93 \pm 0.03$ and the initial output strand concentration $[B]_0 = (7.0 \pm 0.1)\ nM$. The enzyme activity rate constant k_{E} was obtained individually for each trace (Figure S18). To predict the reaction half-life times as function of blocker and enzyme concentration (Figure 5c,e), we applied the average value of the activity rate constants for each set ($k_{\text{E1}} = (5.4 \pm 0.5) \cdot 10^{-4}/s$ and $k_{\text{E2}} = (3.0 \pm 0.3) \cdot 10^{-4}/s$, see Figure S18).

3.Supplementary figures

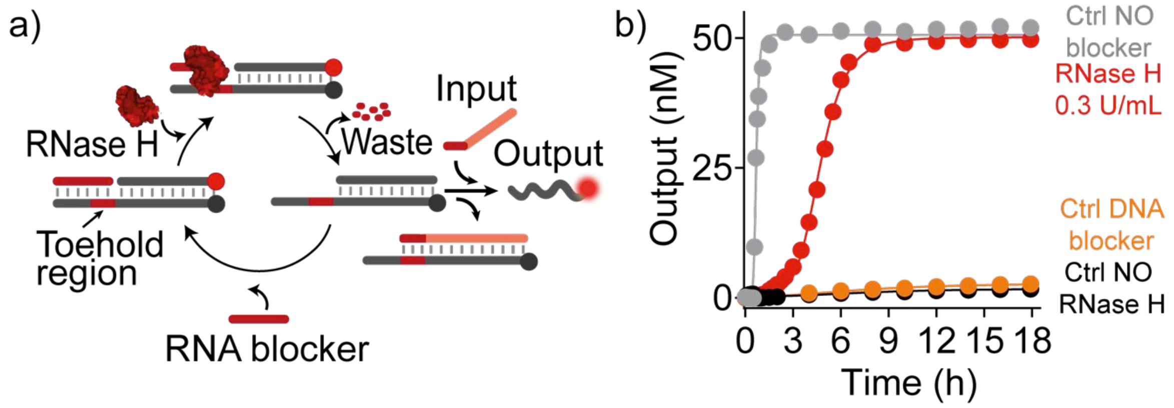

Figure S1. Delayed DNA strand displacement using RNA-blocker and RNase H. a) Scheme of the RNase H-mediated reaction. b) Time-course experiments of the SDR carried out in the following conditions: i) without blocker strand (grey trace), ii) with blocker strand and RNase H (red trace), iii) with blocker strand and without RNase H (black trace); iv) with a DNA blocker and RNase H (orange trace). Shown experiments were performed in Tris HCl 20 mM, $MgCl_2$ 10 mM, EDTA 1mM; pH 8.0 at T=30°C. [Target duplex] = 50 nM, [input strand] = 50 nM, [RNA/DNA blocker] = 150 nM.

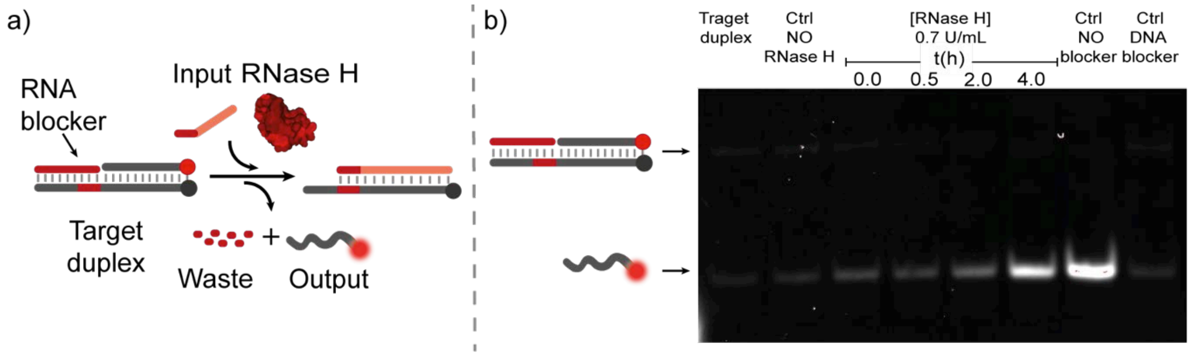

Figure S2. Gel electrophoresis of delayed RNase H-based DNA strand displacement reactions. a) Scheme of reaction. b) Gel electrophoresis experiments confirm the delay observed with fluorescent time-course experiments (Figure 2). In the absence of the enzyme (Ctrl NO RNase H) the reaction does not proceed. In the absence of the blocker strand (Ctrl NO blocker) the reaction proceeds to completion within 30 minutes. Control using a DNA blocker (Ctrl DNA blocker) shows that no reaction is observed even after the addition of the RNase H enzyme. Experimental conditions used here are the same as in Figure 2.

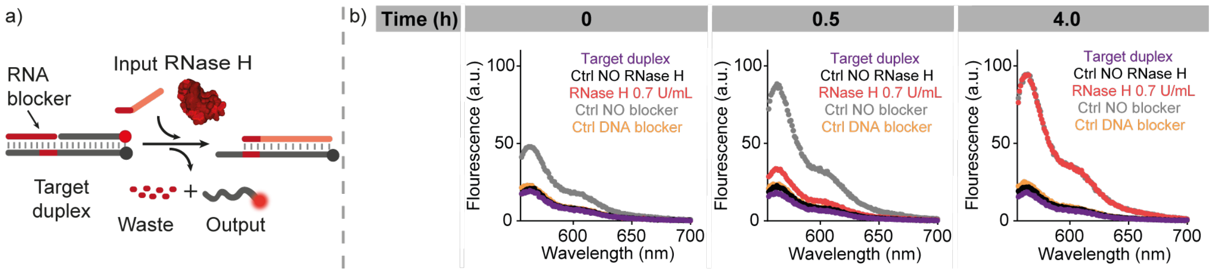

Figure S3. Fluorescence emission spectra of the delayed RNase H-based DNA strand displacement reactions. a) Scheme of the reaction studied. b) Fluorescent spectra at 3 representative times for the same reactions and controls showed in Figure S2.

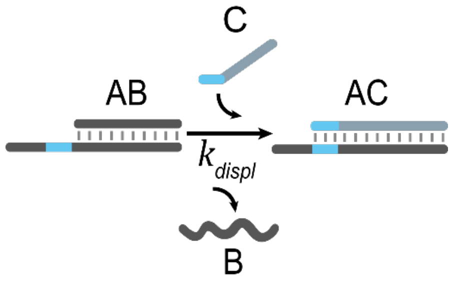

Figure S4. Scheme of a conventional DNA-based strand displacement reactions. An input strand (C) binds to the toehold region (light blue) of the target strand (A) and displaces the pre-hybridized output strand (B).

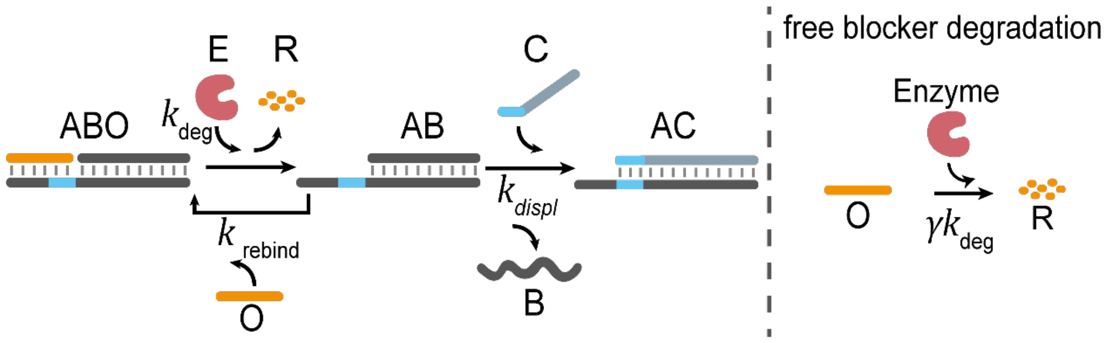

Figure S5. Simple kinetic model for the enzyme-driven delay DNA-based strand displacement reactions. Initially, the blocker strand (O) is bound to the toehold region of the target-output duplex (AB). Degradation of the blocker liberates the toehold, which is then either rebound by a new blocker strand or enables the input (C) to carry out the strand displacement reaction to release the output strand. In parallel, the enzyme may also degrade the unbound single-stranded blocker in solution.

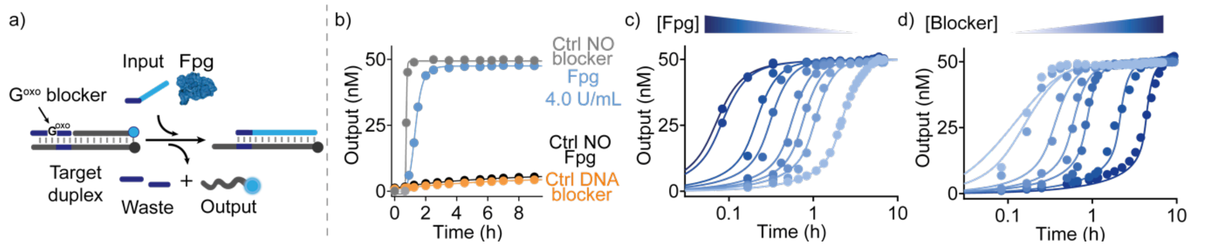

Figure S6. Delayed DNA strand displacement using modified DNA blockers and Fpg enzyme. a) Scheme of the Fpg-mediated reaction. b) Time-course experiments of the SD reaction carried out in the following conditions: i) without blocker strand (grey trace), ii) with blocker strand and Fpg (light blue trace), iii) with blocker strand and without Fpg (black trace); iv) with a DNA blocker and Fpg (orange trace). c) Kinetic traces of strand displacement reaction in presence of G^{oxo}-blocker (150 nM) and varying concentrations of Fpg. d) Kinetics traces of strand displacement reactions in presence of Fpg (8.0 U/mL) and varying concentrations of G^{oxo}-blocker strand. Shown experiments were performed in Tris HCl 20 mM, $MgCl_2$ 10 mM, EDTA 1mM; pH 8.0 at T=30°C. [Target duplex] = 50 nM, [input strand] = 50 nM. In panel b blocker strand concentration was 150 nM.

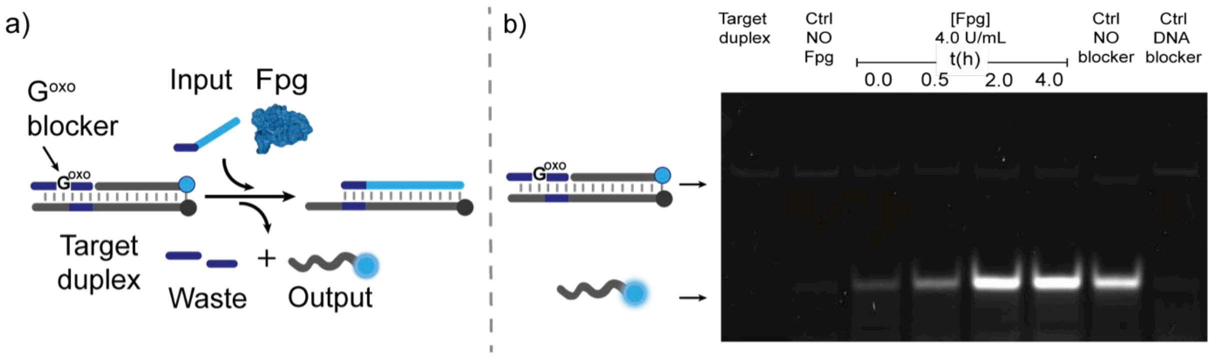

Figure S7. Gel electrophoresis experiments of delayed Fpg-based DNA strand displacement reactions. a) Scheme of the Fpg-mediated reaction. b) Gel electrophoresis experiments confirm the delay observed with fluorescent time-course experiments (Fig. 3). In the absence of the enzyme (Ctrl NO Fpg) the reaction does not proceed. In the absence of the blocker strand (Ctrl NO blocker) the reaction proceeds to completion within 30 minutes. Control using a DNA blocker (Ctrl DNA blocker) shows that no reaction is observed even after the addition of the Fpg enzyme. Experimental conditions used here are the same used in Figure 3.

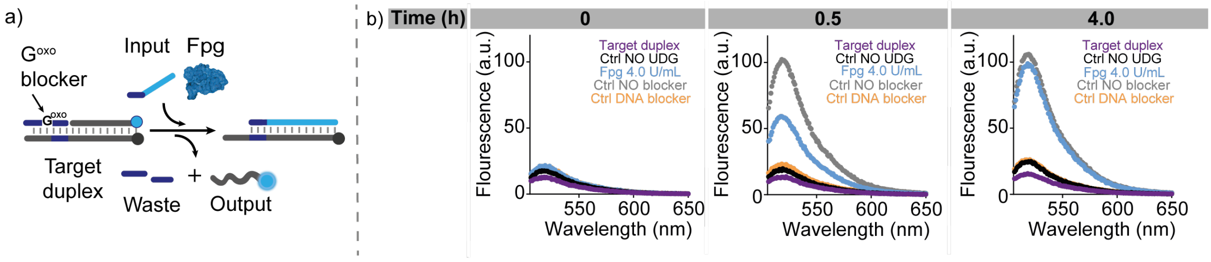

Figure S8. Fluorescence emission spectra of the delayed Fpg-based DNA strand displacement reactions. a) Scheme of the reaction studied. b) Fluorescent spectra at 3 representative times for the same reactions and controls showed in Figure S7.

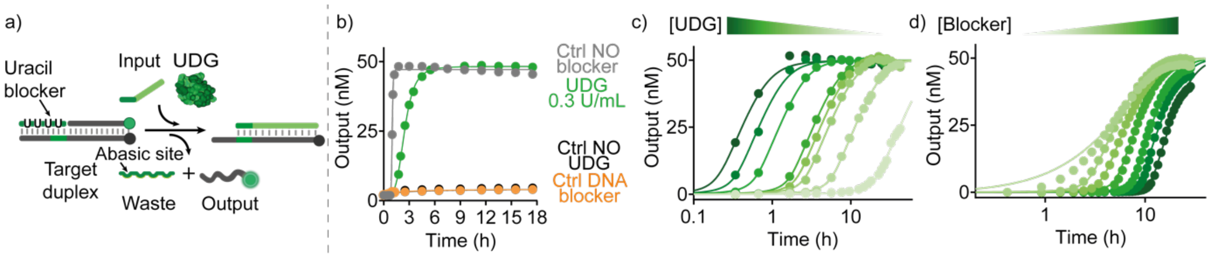

Figure S9. Delayed DNA strand displacement using modified DNA blockers and UDG enzymes. a) Scheme of the UDG-mediated reaction. b) Time-course experiments of the SD reaction carried out in the following conditions: i) without blocker strand (grey trace), ii) with blocker strand and UDG (light blue trace), iii) with blocker strand and without UDG (black trace); iv) with a DNA blocker and UDG (orange trace). c) kinetics traces of strand displacement reaction in presence of uracil-blocker (150 nM) and varying concentrations of UDG. d) kinetics traces of strand displacement reactions in presence of UDG (0.05 U/mL) and varying concentrations of uracil-blocker strand. Shown experiments were performed in Tris HCl 20 mM, $MgCl_2$ 10 mM, EDTA 1mM; pH 8.0 at T=30°C, [target duplex] = 50 nM, [input strand] = 50 nM. In panel b blocker strand concentration was 150 nM.

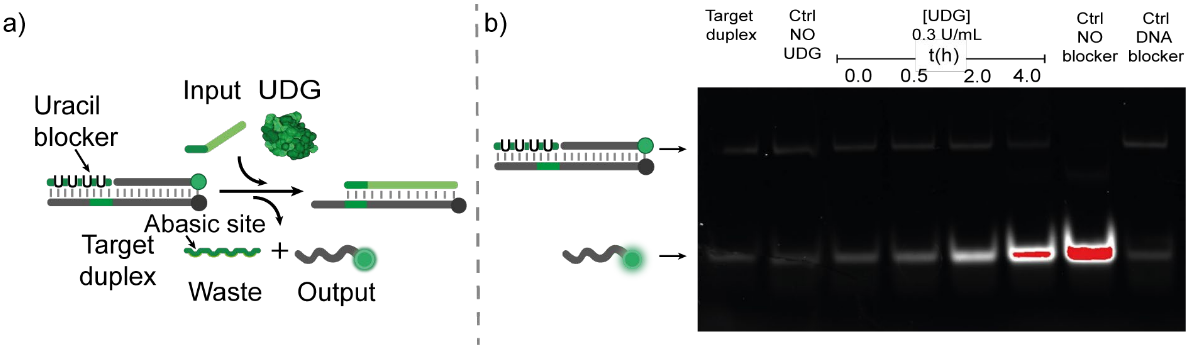

Figure S10. Gel electrophoresis experiments of delayed UDG-based DNA strand displacement reactions. a) Scheme of reaction. b) Gel electrophoresis experiments confirm the delay observed with fluorescent time-course experiments (Fig. 3). In the absence of the enzyme (Ctrl NO UDG) the reaction does not proceed. In the absence of the blocker strand (Ctrl NO blocker) the reaction proceeds to completion within 30 minutes. Control using a DNA blocker (Ctrl DNA blocker) shows that no reaction is observed even after the addition of the UDG enzyme. Experimental conditions used here are the same used in Figure 3.

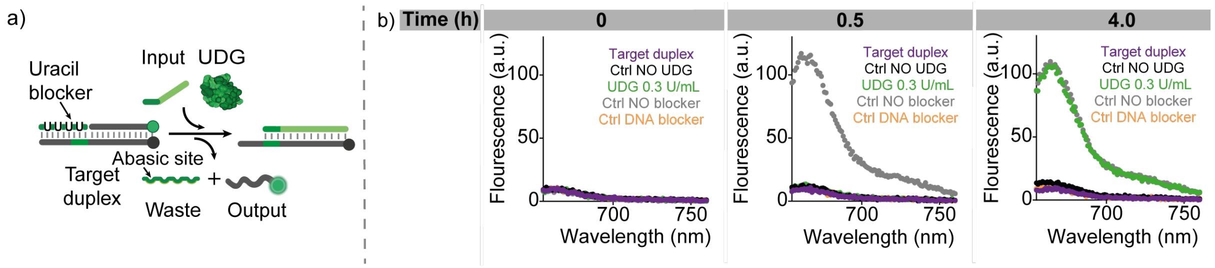

Figure S11. Fluorescence emission spectra of the delayed UDG-based DNA strand displacement reactions. a) scheme of the reaction studied. b) Fluorescent spectra at 3 representative times for the same reactions and controls showed in Figure S9.

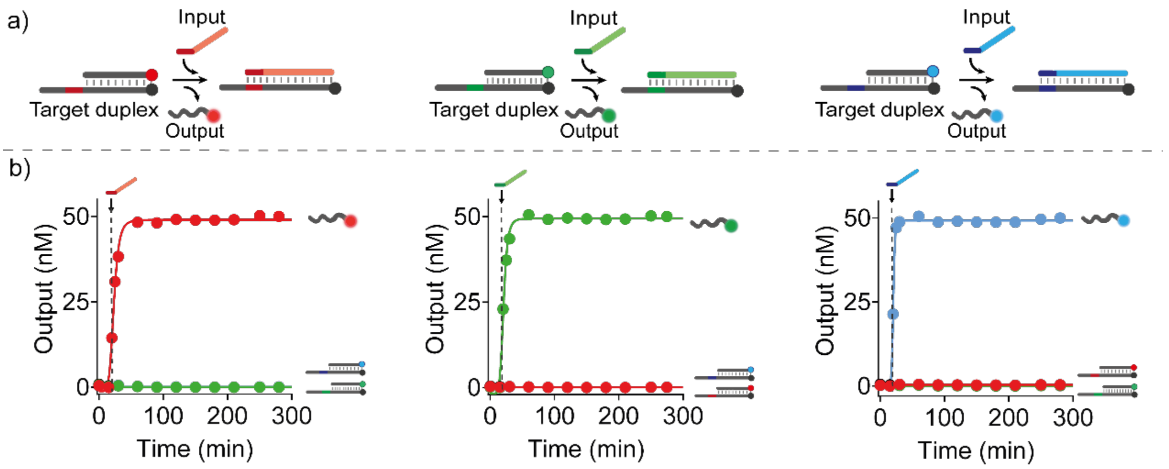

Figure S12. **Orthogonality of the three strand displacement systems probed in absence of blocker.** a) Scheme showing the three different sequence sets: RNase H (red, left), Fpg (light blue, center) and UDG system (green, right), each controlled by a different input strand and each labeled with a different fluorophore/quencher pair for orthogonal temporal control in the same solution. b) Strand displacement reactions each containing the three different target duplexes while being initiated with only a single input. Multicolor fluorescence allows to probe the progress of the displacement reaction of the systems in parallel. Displacement is only obtained for the system with the corresponding specific invader demonstrating full orthogonality. Experiments were performed in Tris HCl 20 mM, $MgCl_2$ 10 mM, EDTA 1mM; pH 8 at T=30°C. [Target duplex] = 50 nM, [RNA-blocker] = 150 nM, [uracil-blocker] = 150 nM, [G^{oxo}-blocker] = 250 nM.

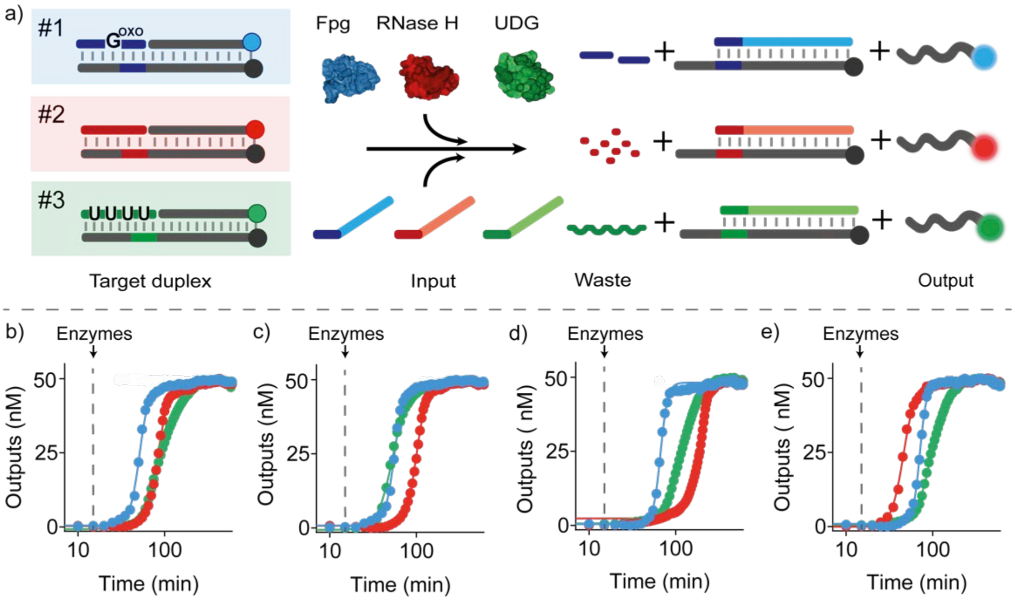

Figure S13. Additional examples of orthogonal temporal control of strand displacement reactions leading to differential activation of the three systems (analogous to Figure 4). a) Scheme showing the three orthogonal systems each controlled by a different enzyme/blocker couple and each labeled with a different fluorophore/quencher pair for orthogonal temporal control in the same solution. b,c,d,e) examples of time-course experiments performed in one solution. Varying the enzyme concentrations, shifts the order of activation of the three systems. Experiments were performed in Tris HCl 20 mM, $MgCl_2$ 10 mM, EDTA 1mM; pH 8 at T=30°C. [Target duplex] = 50 nM, [RNA-blocker] = 150 nM, [uracil-blocker] = 150 nM, [G^{oxo}-blocker] = 250 nM. The following concentrations of enzymes were employed in the time-course experiments: b) Fpg 10.0 U/mL; RNase H 0.7 U/mL; UDG 0.1 U/mL; c) Fpg 10.0 U/mL; RNase H 0.5 U/mL; UDG 0.3 U/mL; d) Fpg 10.0 U/mL; RNase H 0.07 U/mL; UDG 0.3 U/mL; e) Fpg 10.0 U/mL; RNase H 2.0 U/mL; UDG 0.1 U/mL;

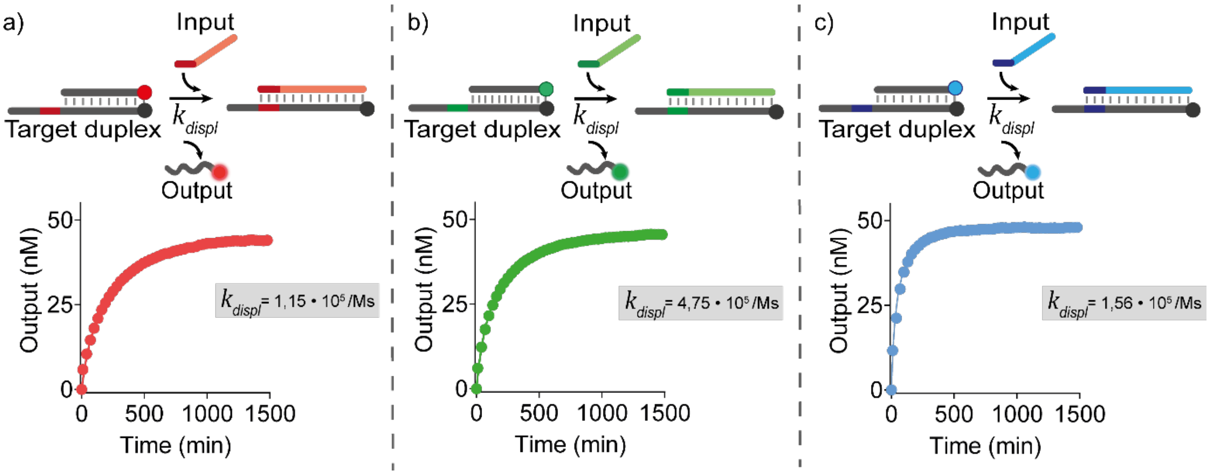

Figure S14. **Conventional strand displacement in absence of blockers**. a,b,c) Time traces (filled circles) of the strand displacement reactions of the different sequence sets (RNase H, Fpg and UDG system, from left to right). After the addition of the specific input strand the reactions occur without delay. Solid lines show fits to the data based on second order kinetics. Experiments were performed in Tris HCl 20 mM, $MgCl_2$ 10 mM, EDTA 1mM; pH 8 at T=30°C. [target duplex] = 50 nM, [input strand] = 50 nM (for each system).

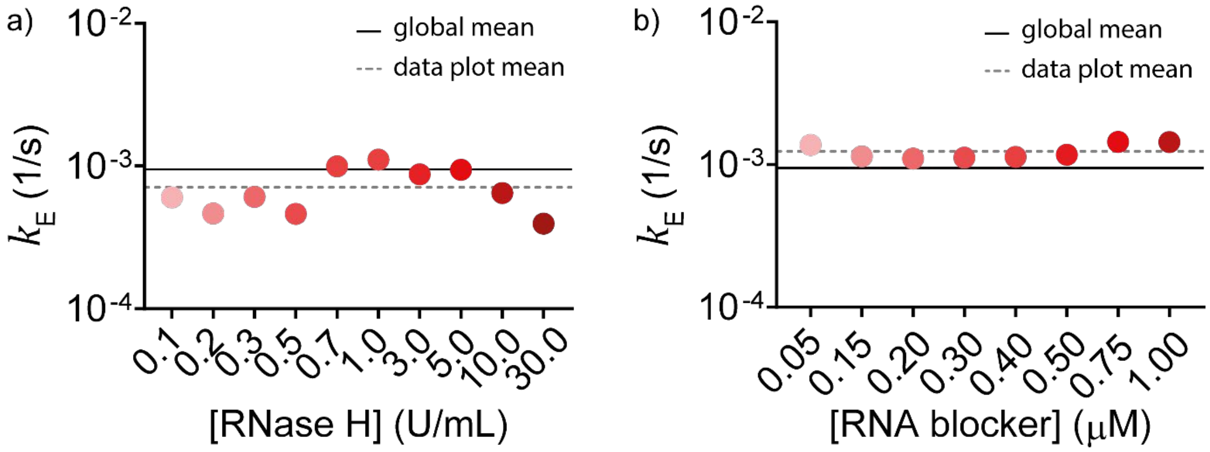

Figure S15. Enzyme activity rate constants for the RNase H system. Rate constants k_E were obtained from the individual traces from a global fit to the data in Figures 2c,e. (a) k_E values for RNase H concentration dependence. (b) k_E values for the blocker concentration dependence. Dashed lines indicate the mean value for the particular measurement set, while the solid lines indicate the mean for both sets. The absence of a trend of k_E with respect to the corresponding concentration suggests that pipetting/mixing errors were mainly responsible for the rate variations.

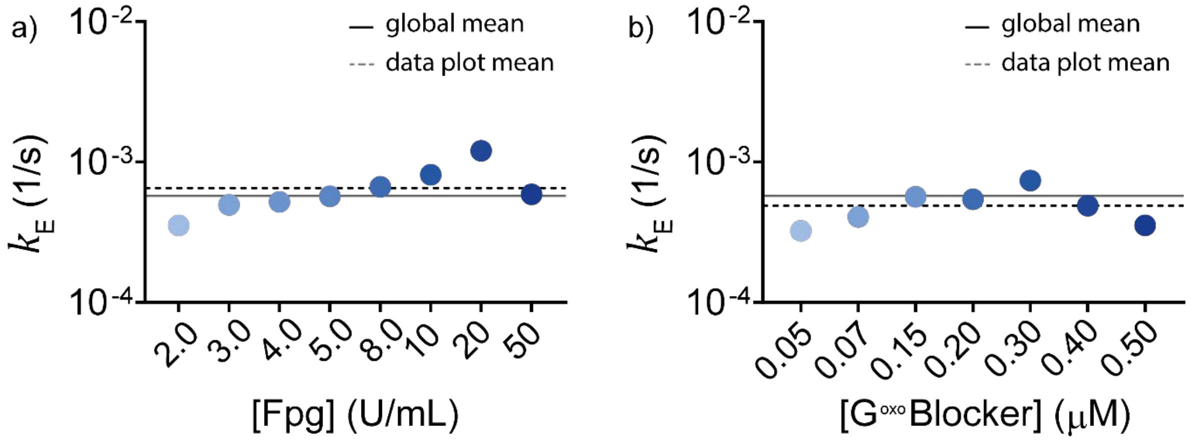

Figure S16. Enzyme activity rate constants for the Fpg system. Rate constants k_E were obtained from the individual traces from a global fit to the data in Figures S6b,c. (a) k_E values for Fpg concentration dependence. (b) k_E values for the blocker concentration dependence. Dashed lines indicate the mean value for the particular measurement set, while the solid lines indicate the mean for both sets. The absence of a trend of k_E with respect to the corresponding concentration suggests that pipetting/mixing errors were mainly responsible for the rate variations.

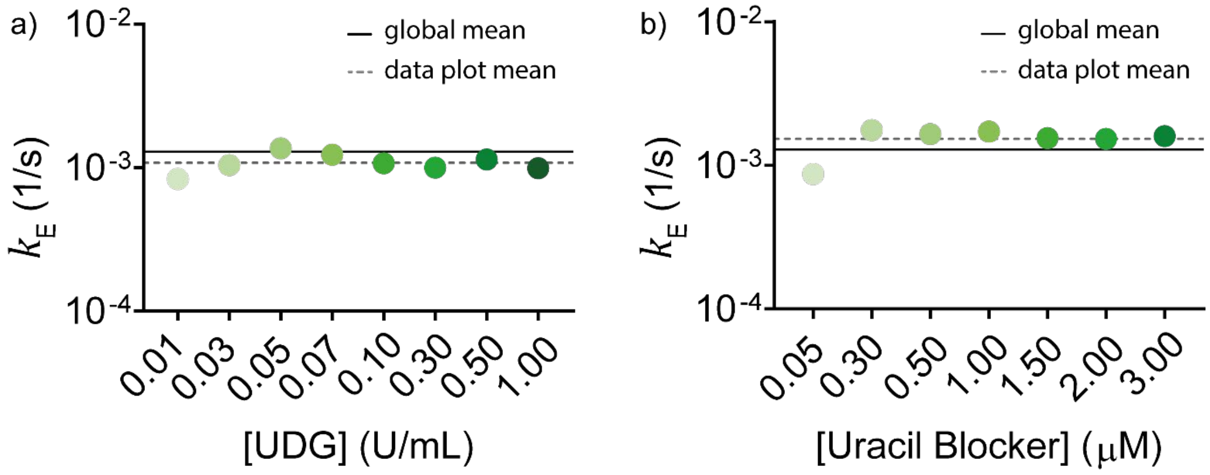

Figure S17. Enzyme activity rate constants for the UDG system. Rate constants k_E were obtained from the individual traces from a global fit to the data in Figures S9e,f. (a) k_E values for UDG concentration dependence. (b) k_E values for the blocker concentration dependence. Dashed lines indicate the mean value for the particular measurement set, while the solid lines indicate the mean for both sets. The absence of a trend of k_E with respect to the corresponding concentration suggests that pipetting/mixing errors were mainly responsible for the rate variations.

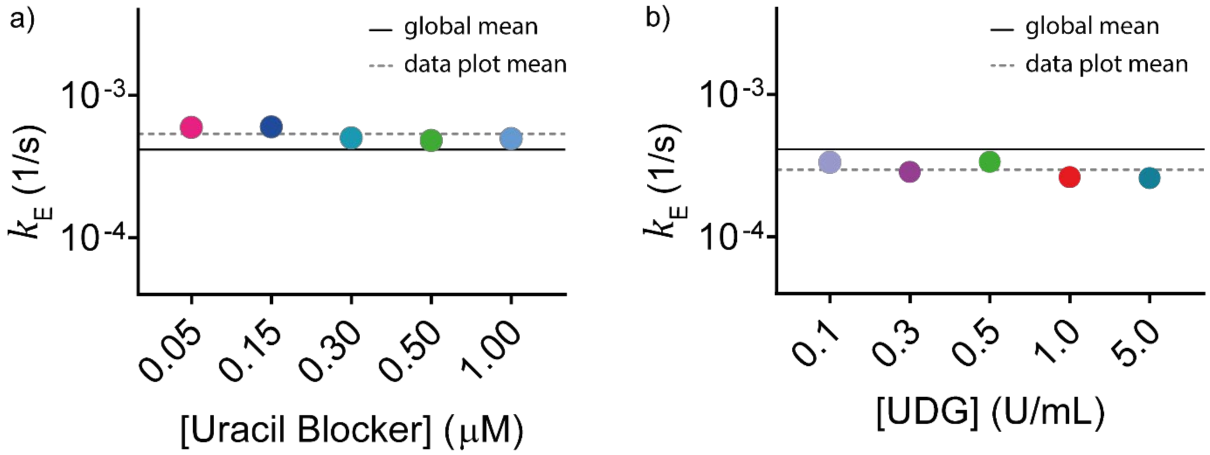

Figure S18. Enzyme activity rate constants for the delayed DNA cargo release from a DNA receptor. Rate constants k_E were obtained from the individual traces from a global fit to the data in Figures 5b,d. (a) k_E values for UDG concentration dependence. (b) k_E values for the blocker concentration dependence. Dashed lines indicate the mean value for the particular measurement set, while the solid lines indicate the mean for both sets. The absence of a trend of k_E with respect to the corresponding concentration suggests that pipetting/mixing errors were mainly responsible for the rate variations.

References

1. Grosso, E. del *et al.* Dissipative Control over the Toehold-Mediated DNA Strand Displacement Reaction. *Angewandte Chemie International Edition* e202201929 (2022) doi:10.1002/ANIE.202201929.

2. Virtanen, P. *et al.* SciPy 1.0: fundamental algorithms for scientific computing in Python. *Nature Methods 2020 17:3* **17**, 261–272 (2020).

3. Ishchenko, A. A., Koval, V. v., Fedorova, O. S., Douglas, K. T. & Nevinsky, G. A. Structural Requirements of Double and Single Stranded DNA Substrates and Inhibitors, Including a Photoaffinity Label, of Fpg Protein From Escherichia Coli. *http://dx.doi.org/10.1080/07391102.1999.10508363* **17**, 301–310 (2012).

4. Panayotou, G., Brown, T., Barlow, T., Pearl, L. H. & Savva, R. Direct Measurement of the Substrate Preference of Uracil-DNA Glycosylase. *Journal of Biological Chemistry* **273**, 45–50 (1998).

5. Bellamy, S. R. W. & Baldwin, G. S. A kinetic analysis of substrate recognition by uracil-DNA glycosylase from herpes simplex virus type 1. *Nucleic Acids Research* **29**, 3857–3863 (2001).
